# Microevolution in the major outer membrane protein OmpA of *Acinetobacter baumannii*

**DOI:** 10.1099/mgen.0.000381

**Published:** 2020-06-04

**Authors:** Alejandro M. Viale, Benjamin A. Evans

**Affiliations:** ^1^​ Instituto de Biología Molecular y Celular de Rosario (IBR, CONICET) and Departamento de Microbiología, Facultad de Ciencias Bioquímicas y Farmacéuticas, Universidad Nacional de Rosario (UNR), 2000 Rosario, Argentina; ^2^​ Norwich Medical School, University of East Anglia, Norwich, UK

**Keywords:** *Acinetobacter baumannii*, outer membrane protein, OmpA, protein evolution, recombination

## Abstract

*
Acinetobacter baumannii* is nowadays a relevant nosocomial pathogen characterized by multidrug resistance (MDR) and concomitant difficulties to treat infections. OmpA is the most abundant *A. baumannii* outer membrane (OM) protein, and is involved in virulence, host-cell recognition, biofilm formation, regulation of OM stability, permeability and antibiotic resistance. OmpA members are two‐domain proteins with an N‐terminal eight‐stranded β‐barrel domain with four external loops (ELs) interacting with the environment, and a C‐terminal periplasmic domain binding non‐covalently to the peptidoglycan. Here, we combined data from genome sequencing, phylogenetic and multilocus sequence analyses from 975 strains/isolates of the *Acinetobacter calcoaceticus */* Acinetobacter baumannii* complex (ACB), 946 from *A. baumannii *, to explore *ompA* microevolutionary divergence. Five major *ompA* variant groups were identified (V1 to V5) in *A. baumannii *, encompassing 52 different alleles coding for 23 different proteins. Polymorphisms were concentrated in five regions corresponding to the four ELs and the C‐terminal end, and provided evidence for intra‐genic recombination. *ompA* variants were not randomly distributed across the *A *. *baumannii* phylogeny, with the most frequent V1(lct)a1 allele found in most clonal complex 2 (CC2) strains and the second most frequent V2(lct)a1 allele in the majority of CC1 strains. Evidence was found for assortative exchanges of *ompA* alleles not only between separate *A *. *baumannii* lineages, but also different ACB species. The overall results have implications for *A. baumannii* evolution, epidemiology, virulence and vaccine design.

## Data Summary

All of the data in this study was downloaded from public databases. A complete list of the GenBank assembly accession numbers, or PubMLST id numbers, for all of the *Acinetobacter* genome sequences used in this study are given in Table S6 (available in the online version of this article). Accession numbers for other bacterial species are provided in Table S5. Accession numbers for specific contigs containing *ompA* alleles are given in Table S1, and accession numbers for non‐*A. baumannii Acinetobacter* genomes containing specific *ompA* alleles are given in Table S2.

**Impact Statement**

*
Acinetobacter baumannii* is an increasing MDR threat in nosocomial settings associated with prolonged hospitalization and concomitantly increased healthcare costs. The main *A *. *baumannii* OM protein, OmpA, is a multifaceted two‐domain protein implicated in host-cell recognition and adhesion, cytotoxicity, biofilm formation and as a slow porin for antibiotics and small hydrophilic nutrients. *A. baumannii* OmpA has been proposed as a potential target for anti‐virulence drugs and as a vaccine candidate. Given the many interactions of this protein with environmental factors including host defenses, it is certainly subjected to many selective pressures. Here, we analysed the microevolution of this OM protein in the *A *. *baumannii* population to obtain clues on the extent to which selection in the clinical setting has shaped this protein. The results provide relevant information on the main causes driving evolution of this protein, with potential implications in *A. baumannii* epidemiology, virulence and vaccine design. Vaccines are being explored as a potential new approach to combat bacterial pathogens that have become resistant to most or all antibiotics available to treat them. *A. baumannii* is one such WHO priority pathogen, and OmpA proposed as a vaccine target. Many recent studies have investigated the virulence properties associated with OmpA and its immunogenicity. However, these studies do not take into account the large degree of variation in OmpA sequence we describe here, which is concentrated in the protein regions that would act as antigenic determinants. Therefore, our work illustrates the general principle that a detailed understanding of the diversity and evolution of proposed antigens is essential for the efficient and effective development of vaccines against MDR bacterial pathogens.

## Introduction

The genus *Acinetobacter* (family *Moraxellaceae *, class *Gammaproteobacteria *) is composed of Gram‐negative bacterial organisms of ubiquitous environmental distribution [1]. It includes a number of phylogenetically closely related species generally associated with the human environment grouped into the so‐called *Acinetobacter calcoaceticus */* Acinetobacter baumannii* complex (ACB). Of these, *A. baumannii* represents the most problematic nosocomial species generally affecting critically ill patients [1–7]. Molecular-typing studies have indicated that infective *A. baumannii* strains belong to a limited number of globally distributed clonal groups/complexes (CCs) estimated to have emerged and disseminated after 1960, and from which CC2 and CC1 have been the most thoroughly characterized [2–5, 7–10]. The reasons for the success of CC strains as human pathogens are still poorly understood, but are thought to include the general resilience of the species, an increased ability to colonize the human environment, and the propensity to acquire exogenous genetic material [2–7, 10]. Thus, most CC strains not only show multi‐drug resistance (MDR) phenotypes from the acquisition of different mobile genetic elements enriched in resistance genes, but can also rapidly modify virulence‐related surface structures recognized by host defenses as the result of recombination events involving discrete genomic loci [2–5, 7, 10–13]. This has left very few treatment options available [2–5, 7, 11, 14, 15] and has led to the alternative search of targets for anti‐virulence drugs or vaccines, and in this context a potential candidate is represented by the major *A. baumannii* outer membrane (OM) protein, OmpA [5, 6, 16–24]. *A. baumannii* OmpA belongs to the widely distributed OmpA/OprF family of OM proteins from which *Escherichia coli* OmpA and *Pseudomonas* OprF are well‐characterized examples [25–27]. These proteins are structured in two domains: an N‐terminal eight‐stranded β‐barrel embedded in the OM exposing four loops to the environment (ELs), and a C‐terminal periplasmic domain that binds the peptidoglycan and contributes to cell-envelope stability [26–28]. *E. coli* OmpA ELs play important roles in target-cell recognition and bacterial survival once internalized, and variations in EL2 and EL3 composition or length affect the invasive potential of the bacterial cells [26]. Given the many interactions of ELs with environmental factors, it is not surprising that they represent the less conserved protein regions with both mutation and recombination providing for the observed variabilities [25–27, 29]. *A. baumannii* OmpA has been implicated in a multitude of functions that range from structural roles, a slow porin for small hydrophilic nutrients and antimicrobials, and virulence including fibronectin binding, biofilm formation, adhesion to host cells and cytotoxicity [3, 5, 6, 20, 21, 27, 29–37]. It has also been shown to interact with host defenses, inducing innate immune responses, the production of antibodies both in *A. baumannii *‐infected patients and experimentally infected mice, and to bind antimicrobial peptides via the ELs [17–24, 38]. It follows that there are different selective pressures including trade‐off mechanisms [26, 27, 39–43] acting on *A. baumannii* OmpA. However, to our knowledge no systematic studies have been conducted on *A. baumannii* OmpA microevolution. Here, we analysed the polymorphism of *A. baumannii* OmpA and the roles of mutation and recombination in defining this variability, to analyze the extent to which selection in the clinical setting has shaped this protein.

## Methods

### Sequence retrieval, alignments and phylogeny estimations

**Fig. 1.**
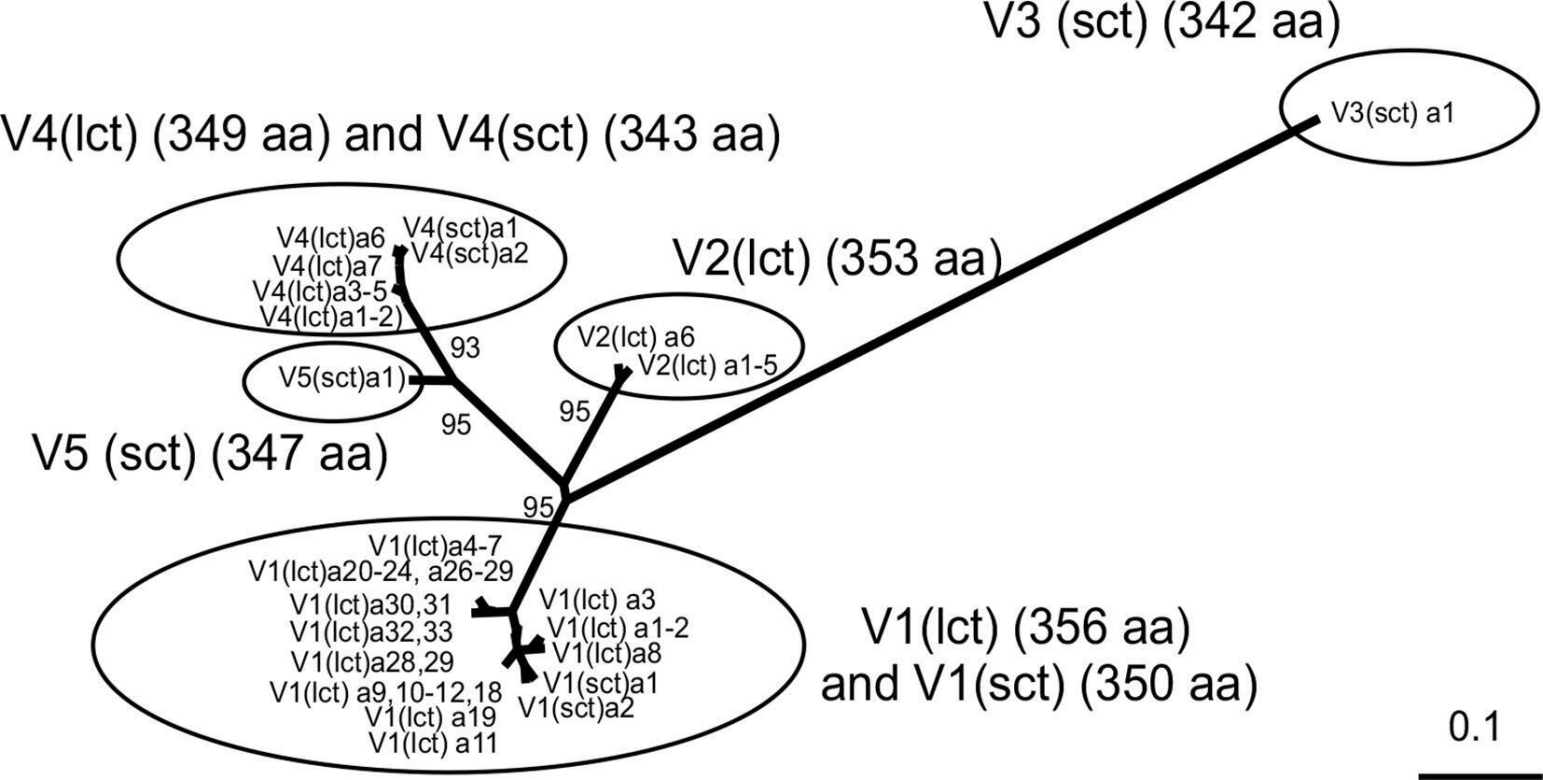
Co‐existence of different OmpA variants and sub‐variants in the *A. baumannii* population. An unrooted ML phylogenetic tree was constructed using PhyML (http://phylogeny.lirmm.fr/phylo_cgi/index.cgi) using alignments of translated *ompA* sequences representing all OmpA protein alleles identified in *A. baumannii* in this work. The lengths of the branches are proportional to the evolutionary distance, with the scale bar (estimated changes per site) shown at the bottom right. Bootstrap support (percentages of 100 re‐samplings) for the different clusters are indicated at the corresponding branches. The analysis shows that OmpA proteins found in *A. baumannii* comprise five well‐defined similarity groups of alleles (V1–V5, indicated by ovals) each defining a particular variant. Some variants such as V1 and V4 in *A. baumannii* additionally encompass a sub‐variant group identified with the suffix (lct) or (sct) (implying longer C‐terminal tract and shorter C‐terminal tract, respectively) that differ between them by the presence/absence of a hydrophobic tract of six amino acid residues composed mostly by alanine residues close to the corresponding C‐terminal ends (see the main text for a more detailed description). The lengths in amino acid residues of the corresponding variant and sub‐variant OmpA proteins are indicated for each variant cluster.

The nucleotide sequence of *ompA* from *A. baumannii* ACICU (accession number NC_017162.1, locus tag ABK1_RS15935) was used to query the JGI SIMG/M database (https://img.jgi.doe.gov/) for available *ompA* sequences from *A. baumannii* genomes. *ompA* nucleotide sequences obtained by this procedure were translated, aligned, separated into five groups of similarity on the basis of a maximum‐likelihood (ML) phylogenetic analysis (Fig. 1, see below). A representative sequence from each group was used as a query to search for homologous *ompA* sequences in the strains/isolates present in the *A. baumannii* MLST (Pasteur) database (updated 14 January 2020; 1256 sequences) from which whole-genome sequence data were additionally available (https://pubmlst.org/bigsdb?db=pubmlst_abaumannii_pasteur_seqdef). The blastn data analysis plugin tool provided in the database was used for this purpose. *ompA* sequences identified in each of the strains/isolates were then extracted using the tools also provided in the database, and assigned to a given variant/allele group. The sequence identification numbers (Seqbin id#:) as well as the nucleotide initiation and termination numbers covered by the corresponding *ompA* sequences extracted using MLST database tools are provided for each strain/isolate in Table S1. For a small number of *A. baumannii* isolates, which received ST assignments but lacked genome sequence information in the MLST database, but for which complete genome, scaffolds or contig sequences were available from the NCBI database (https://www.ncbi.nlm.nih.gov/genome/genomes/403?), *ompA* sequences were extracted from this database using the blastn tool and incorporated into the analysis conducted here. In these cases the corresponding sequence accession numbers and location of the *ompA* nucleotide sequences are also provided in Table S1. Also, for selected *A. baumannii* isolates collected over a 30‐year period (Table S1) and characterized as belonging to lineage 1 (L1) of global clone 1 (GC1) [10], the pertinent housekeeping gene sequences were extracted besides the corresponding *ompA* sequences from the sequence data available in the NCBI database. In these cases ST assignments were inferred from these data following comparisons with the corresponding allele sequences of the MLST Pasteur scheme (https://pubmlst.org/bigsdb?db=pubmlst_abaumannii_pasteur_seqdef&page=downloadAlleles). Expectedly, all of these isolates belonged to clonal complex 1 (CC1) (see Table S1 for details).

A similar procedure to that described above was used to detect *ompA* sequences among 29 strains/isolates of non‐*baumannii* ACB species including *A. calcoaceticus *, *A. lactucae *, *A *. *nosocomialis *, *A. oleivorans *, *A. pittii* and *A. seifertii *, for which whole-genome sequence data were available in the NCBI database (https://www.ncbi.nlm.nih.gov/genome/genomes/). In these cases the corresponding accession numbers and the location of the genes in the analysed sequence data are indicated in Table S2.

*A. baumannii* strains/isolates in which incomplete *ompA* sequences resulting from premature termination codons, frameshifts, and/or insertions as detected by the sequence comparison analyses conducted in this work were omitted from the analysis. In the case of redundant isolates, only one was included in the analysis. Moreover, in the case of V1 and V2 *ompA* alleles found in *A. baumannii* (the most abundant sequences found, Table S2), nucleotide substitutions found only once among all *ompA* sequences analysed were considered sequencing errors and the corresponding isolates were also omitted from the analysis. This left 946 *A. baumannii* strains/isolates (Tables S1 and S2) analysed in this work. Expectedly, the database was biased towards *A. baumannii* clinical isolates of human origin, in particular CC2 and CC1 isolates, which encompassed 53 and 7.7 %, respectively, of the total sample (Table S1). Information provided by the MLST database of an origin other than a human source was incorporated into Table S1.

Protein or nucleotide ML phylogenetic trees were constructed using PhyML (http://phylogeny.lirmm.fr/phylo_cgi/index.cgi). Assembled *Acinetobacter* genomes used in this work were obtained from the NCBI database as detailed above. All genome accession numbers are given in Table S6. A core gene alignment was obtained using Roary [44], and the ML phylogeny was estimated using FastTree v2.1.10, with support estimated from 1000 resamples [45].

### Protein sequence diversity

Variation between *A. baumannii* OmpA variants was determined by calculating the Shannon entropy from alignments of the protein alleles described here (https://www.hiv.gov/content/sequce/ENTROPY/entropy.html), as described previously for *A. baumannii* CarO [11].

### Intragenic recombination detection

An alignment containing one of each *A. baumannii ompA* allele was loaded into the Recombination Detection Program v4 (RDP4) [46]. An automated recombination analysis was run using the RDP [47], GENCONV [48] and Chimera [49] methods. Regions in which significant evidence for recombination was detected were removed using the ‘Save alignment with recombinant regions removed’ feature in RDP, and used in subsequent analyses.

### Tests for selection

To investigate whether natural selection is acting similarly in the *A. baumannii ompA* EL regions as compared to the rest of the protein, we used a partition model in the program codeml implemented in PAML v4.9d [50, 51]. Separate alignments for alleles from groups V1, V2 and V4 either including all data, or with recombinant regions removed, were used to estimate trees using PhyML, which then had distance information manually removed for use as input to codeml. Input alignment files were partitioned into sequences corresponding to exposed EL regions and those corresponding to non‐exposed TM and periplasmic domains. Two models were fitted to the data in codeml, a null model where parameter values are assumed to be equal between the exposed and non‐exposed regions of the sequence (model =0, NSsites=0, Mgene=0), and an alternative model where *κ* (transition/transversion rate ratio), *ω* (*d*N/*d*S), *π*s (codon frequencies) and *c*s (proportional branch lengths) are allowed to vary between the two regions (model=0, NSsites=0, Mgene=4). The resulting log likelihood values from the two models were compared using χ^2^ tests to determine whether the alternative models better fit the data than the null models. V3 and V5 were excluded from this analysis as they only contain one allele each in *A. baumannii *.

## Results

### OmpA polymorphism in the *A. baumannii* population

**Fig. 2.**
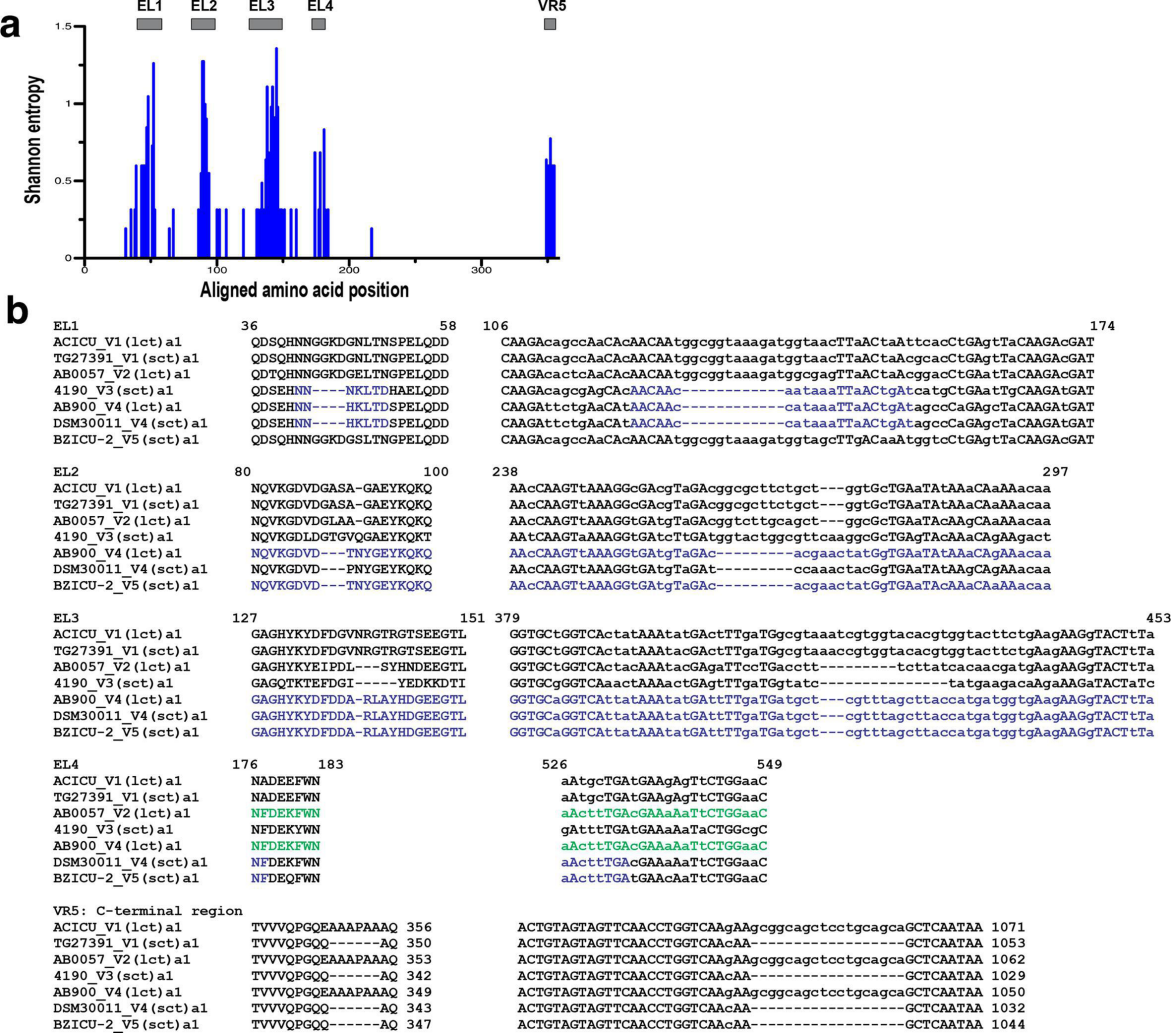
Sequence variability between *A. baumannii* OmpA variants and sub‐variants. (a) Shannon-entropy variation along the amino acid alignments of the OmpA variant and sub‐variant representative alleles shown in Fig. 1. The program available at http://www.hiv.lanl.gov/content/sequence/ENTROPY/entropy.html was used for entropy computations. The heights of the bars are proportional to the degree of amino acid variation at a particular location in the alignments. The span of the predicted external loops EL1 to EL4 at the N‐terminal domain and the variable C‐terminal motif (VR5) are indicated by closed bars above the figure. Alignments are numbered from the corresponding N‐terminal regions including the signal peptide. (b) Amino acid (left columns) and corresponding nucleotide alignments (right columns) of the EL regions and the C‐terminal domains of representative *omp*A variant alleles. The amino acid and nucleotide positions encompassing the EL1 to EL4 regions indicated above the sequences are those corresponding to ACICU (V1) OmpA. In VR5 the numbers at the end indicate the position of the last amino acid or nucleotide in the corresponding columns, and the high‐GC 5´‐GCGGCAGCTCCTGCAGCA‐3′ insertion coding for the extra AAAPAA stretch found at the C‐terminus in some variant sequences is shown. Uppercase letters in nucleotide alignments indicate the same base at a given position in all sequences. RDP4 detected evidence for recombination between V3 and V4 variants at EL1 and between V4 and V5 variants at a gene region encompassing the entire EL3 and part of EL4 (highlighted in blue). In addition, visual inspection detected sequence identity between V4 and V5 sequences along the entire EL2 (highlighted in blue), as well as between V2 and V4 along the complete EL4 region [highlighted in green for V2 and V4(lct) only]. The complete amino acid and nucleotide alignments, as well as the topology predictions, are shown in Figs S1 and S2.

*ompA* sequences were extracted from 975 genomes of ACB strains/isolates deposited in databases, of which 946 were *A. baumannii* (Table S1 and Table S2, see Methods for details). A ML phylogeny based upon the alignments of the corresponding translated sequences identified five well‐defined OmpA variant groups, hereafter called V1 to V5 (Fig. 1). In *A. baumannii *, each of the identified variant groups was composed of a different number of alleles sharing high levels of nucleotide sequence identity (>98%) between them (Table S2). Between these different variants the protein sequence identities ranged from ca. 94 % for V1 and V2 to ca. 85 % between them and the most divergent group V3. An unequal distribution of *ompA* variants was found, with almost 95 % of the *A. baumannii* strains/isolates carrying alleles from only V1 and V2 (Tables 1 and S2). Also, *A. baumannii* V1 and V4 variants each encompassed two sub‐variant groups designated V1(lct)/V1(sct) and V4(lct)/V4(sct), respectively (Tables 1 and S1, S2, Figs 2, S1 and S2). Each subgroup could be distinguished by the presence/absence of a six amino acid stretch (AAAPAA) close to the C‐terminal end, thus defining QEAAAPAAAQ (longer C-terminal tract-lct) or QQAQ (*s*horter *C*‐terminal *t*ract ‐ sct) as alternate C‐terminal sequences in the corresponding proteins. The possible origin of this stretch is discussed in more detail below.

**Table 1.** Characteristics of the different *A. baumannii* OmpA variant (V) groups

V group	No. of alleles (genes/ proteins)	No. of *A. baumannii* strains/ isolates	Gene length (nt)	Protein precursor length (aa)	Mature protein length (aa)	C-terminal sequence	% in *A. baumannii* isolates (total=946)
V1(lct)	32/12	723	1071	356	334	QEAAAPAAAQ	76.43 %
V1(sct)	2/2	6	1053	350	328	QQAQ	0.63 %
V2(lct)	6/2	167	1062	353	331	QEAAAPAAAQ	17.63 %
V3(sct)	1/1	20	1029	342	320	QQAQ	2.1 %
V4(lct)	7/4	23	1050	349	327	QEAAAPAAAQ	2.43 %
V4(sct)	2/2	2	1032	343	321	QQAQ	0.21 %
V5(sct)	1/1	5	1044	347	325	QQAQ	0.53 %

The OmpA V1(lct) subgroup was by far the most frequently observed among the *A. baumannii* population analysed, with 76.4 % of all strains/isolates analysed carrying V1(lct) alleles (Tables 1 and S2). It encompassed 32 gene alleles differing by a maximum of 20 nucleotide substitutions, translated to 12 different OmpA proteins differing from none to a maximum of four amino acid substitutions. Among them, the V1(lct)a1 allele was predominant, representing more than 66 % of all V1 alleles detected and being found in 51 % of the total *A. baumannii* strains/isolates analysed (Table S2). Of note, the *A. baumannii* type strain ATCC 19606 and also the ATCC 17978 strain, isolated from human clinical materials around or before 1950 [52, 53], display V1(lct) alleles [V1(lct)a14 and V1(lct)a19, respectively, Table S1]. This dates the association of *ompA* V1(lct) alleles with *A. baumannii* clinical strains prior to the emergence and spread of the main epidemic clones, estimated to have occurred in the 1960s [4, 10]. Our blastn search among genome sequences of non‐*baumannii* ACB species deposited in the NCBI database identified other V1(lct) alleles among a few strains/isolates including *A. pittii* XJ88, *A. pittii* 2012N08‐034, *A. lactucae* CI78 and *A. oleivorans* DR1 sub‐species DE0008 (Table S2). Concerning the occurrence of V1(sct) alleles displaying the QQAQ short-terminal tract, only two alleles were found in a small percentage (0.53 %) of *A. baumannii* strains/isolates (Tables 1 and S2). More divergent V1(sct) alleles were also identified among various non‐*baumannii* ACB species including strains of *A. pittii *, *A. nosocomialis* and *A. seifertii* (Table S2).

V2(lct) was the second most frequent OmpA variant found in *A. baumannii *, being present in almost 18 % of the strains/isolates analysed (Table 1). Six different V2(lct) gene alleles were detected in *A. baumannii* differing in a maximum of two nucleotide substitutions translated to two V2 OmpA proteins displaying one amino acid substitution between them (Table S2). Other and more divergent V2(lct) alleles were detected also in a limited number of non‐*baumannii* ACB species, including *A. calcoaceticus* ANC3811 and *A. oleivorans* strains KCJK7897 and TUM15571 (Table S2).

The OmpA V3 group had a much lower representation (2.1 %) in *A. baumannii* (Table 1), with all strains/isolates displaying a V3(sct) ‘short’ QQAQ C‐terminal sequence type (Tables S1 and S2). Other more divergent V3(sct) alleles were detected among various non*-baumannii* ACB species including different *A. nosocomialis *, *A. calcoaceticus* and *A. pittii* strains (Table S2). Notably, we also detected the presence of a subgroup of V3 alleles carrying the (longer) QEAAAPAAAQ C‐terminal tract [designated V3(lct)] among a number of non*-baumannii* ACB species including the *A. oleivorans* type strain DR1 sub‐species DR1 (https://www.ncbi.nlm.nih.gov/biosample/SAMN02603211) and different strains of *A. pittii *, *A. calcoaceticus* and *A. lactucae* including the *A. lactucae* type strain NRRLB‐41902 (https://www.ncbi.nlm.nih.gov/biosample/SAMN04437545).

The OmpA V4 group also had a low representation in *A. baumannii* (2.6 %, Tables 1, S1 and S2). Most strains/isolates carried the V4(lct) subgroup of alleles, which was composed of seven alleles differing by a maximum of 13 nucleotide substitutions translated to four proteins differing in a maximum of two amino acid substitutions (Table S2). A V4(sct)sub‐group of alleles displaying the (shorter) QQAQ C‐terminal end was also found in two of the *A. baumannii* strains/isolates including the environmental DSM30011 strain isolated before 1944 from a desert plant source [54]. Remarkably, our blastn search failed to detect V4 alleles among non‐*baumannii* ACB species.

Finally, V5 also had a very low representation in *A. baumannii* (0.53 %), with only five strains all showing the same V5(sct)a1 allele (Tables 1 and S2). Among non‐*baumannii* ACB species V5(sct) alleles were also detected among a number of *A. pittii* strains that included the type strain ATCC 19004 (https://www.ncbi.nlm.nih.gov/biosample/SAMN01828154) (Table S2). Notably also, a V5(lct) subvariant displaying QEAAAPAAAQ as the C‐terminal tract was also detected in the *A. pittii* strain WCHAP005046 (Table S2).

### *A. baumannii* OmpA polymorphism

A Shannon-entropy plot of all aligned *A. baumannii* OmpA variant alleles (Fig. 2a) showed that approximately 60 from a total of almost 357 amino acid positions (17 %) are polymorphic. This polymorphism was concentrated in five well‐defined regions, four of them overlapping with the four extracellular loops located at the N‐terminal domain (EL1–EL4) and the fifth at the C‐terminal end (Fig. 2a). Amino acid (Fig. 2a) and nucleotide (Fig. 2b) alignments show that the differences in length among variants are due to indels located in EL1, EL2 and EL3, and in the C‐terminal end.

### Selection acting on *ompA* V1(lct) alleles

V1(lct) *ompA* was by far the most frequent variant found in *A. baumannii *, it encompassed the largest number of different alleles (Table 1), and was also found among non‐*baumannii* ACB species including strains of *A. pittii *, *A. lactucae* and *A. oleivorans* (Table S2). The large differences in frequency between the different V1(lct) alleles and the predominance of V1(lct)a1 (Table S2) led us to examine in more detail the nature of the changes occurring both within *A. baumannii* (intra‐species changes), and also between *A. baumannii* and non*-baumannii* (inter‐species changes) (Table 2, Fig. S3). Within *A. baumannii* V1(lct) alleles (intra‐species changes) the total number of synonymous substitutions was around 3.85‐fold higher than that of non‐synonymous ones, with five out of the seven non‐synonymous substitutions concentrated in the EL‐coding regions (Table 2). A comparative analysis of the substitution patterns in the EL‐coding regions compared to the rest of the gene confirmed the different molecular evolutionary histories between the two (Table S4). Although there was a bias towards the EL regions carrying non‐synonymous substitutions, a detailed analysis of the resulting amino acid changes indicated that they represented conservative substitutions (Fig. S3). Similar conservative changes at the EL regions were also observed inter‐species by comparing *A. baumannii* V1(lct)a1 with the V1(lct) alleles found in *A. pittii *, *A. lactucae* or *A. oleivorans* (Fig. S3). The overall results indicated a strong pressure operating both intra‐ and inter‐species for the maintenance of the V1(lct) OmpA protein structure, including its exposed EL regions. Still, the large predominance of V1(lct)a1 among other V1(lct) alleles (Table S2, see above) argue against all amino acid substitutions accumulating in this allele as completely lacking adaptive significance. In this context, a comparative analysis of all changes detected (Fig. S3) identified a combination of two amino acid substitutions unique to V1(lct)a1. This combination resulted in the presence of two particular amino acid residues in the exposed regions of the protein, namely Ser52 at EL1 and Thr144 at EL3. It is tempting to speculate that the selective advantage provided by this combination represented the basis of the large predominance of V1(lct)a1 in *A. baumannii *.

**Table 2.** Summary of synonymous and non‐synonymous substitutions at polymorphic sites detected between V1(lct) *ompA* alleles found in strains/isolates of the *A. baumannii* population analysed. The 1071 nucleotide positions encompassing 356 codons of the different V1(lct) *ompA* alleles described in this work were aligned, and the mutations resulting in synonymous (syn) and non‐synonymous (non‐syn) changes were calculated. The numbers between brackets denote the corresponding percentages among the 356 codons in each case

Gene regions	Total changes	Synonymous changes	% syn	Non-synonymous changes	% non-syn
Polymorphic sites found between the 32 *A. baumannii* V1(lct) alleles					
All	34 (9.55)	27 (7.58)	79.4	7 (1.97)	20.6
EL regions	15 (4.21)	10 (2.81)	29.4	5 (1.40)	14.7
Non‐exposed regions (total)	19 (5.34)	17 (4.76)	50	2 (0.56)	5.9
TM regions only	9 (2.53)	9 (2.53)	33.3	0	0

### Intra-genic recombination at the *ompA* locus

Detailed comparative analyses of the amino acid and corresponding nucleotide sequences spanning the *A. baumannii* OmpA variant’s variable regions (Fig. 2b) suggested a history of intra‐genic recombination. The use of RDP4 (46) found evidence for intra‐genic recombinatorial exchanges between a number of alleles from various different V groups (Table S7). Visual inspection additionally detected substantial sequence identity between V4 and V5 along the entire EL2 and between V2 and V4 along EL4 (Fig. 2b). These observations are suggestive of several intra‐genic recombinatorial exchanges during the early stages of evolution of the different *ompA* variants.

Intra‐genic recombination may also explain the existence of two alternate C‐terminal ends among otherwise very similar OmpA variants (see above). The main difference between sub‐ variants resides in the presence/absence of a hydrophobic tract of six amino acids, composed mostly by alanine residues, close to their C‐terminal ends (Fig. 2b, Table S3). We noted that the DNA sequence coding this tract, 5′‐GCGGCAGCTCCTGCAGCA‐3′, shows a higher GC content (72%) when compared to the rest of the *A. baumannii ompA* gene (42%) or even to the average GC content of *Acinetobacter* genomes [1–5, 7]. This suggests that the longer QEAAAPAAAQ C‐terminal region may have derived from a non‐homologous (illegitimate) recombination event involving the insertion of a fragment originating in a high‐GC DNA source outside the *Acinetobacter* genus [55–58]. Moreover, this event is likely to have been selected recently in *A. baumannii* (or in a related ACB complex species), as judged by the presence of OmpAs bearing QEAAAPAAAQ as C‐terminal ends only among ACB members but not the other ecologically differentiated clades (Table S3) that compose the *Acinetobacter* genus [1].

We analysed possible donor(s) of the 18‐nucleotide high‐GC fragment among GenBank bacterial genomes by conducting a blastn search adjusted for short sequences identification (https://blast.ncbi.nlm.nih.gov). Among *Acinetobacter* sequences this stretch was found only in genomes of *A. baumannii* and some other ACB species, always inserted at the same position near the 3′‐end of the *ompA* gene (Fig. 2a, Table S3). Remarkably however, identical or highly similar sequences (17 out of 18 nucleotides identity), which were not linked to *ompA*/*oprF*‐related genes, were also found in the genomes of several bacterial species displaying GC contents ≥60 % and assigned to different phyla including the *Actinobacteria *, the *Bacteroidetes *, and different subdivisions of the *Proteobacteria* (Table S5). Of note, the above taxa provide not only for the predominant micro-organisms in soils but also for the bacterial community associated with hand palms in humans [59]. These interconnecting niches, also shared by *Moraxellaceae* members including *A. baumannii* and other ACB species [1, 59], comprise a gene exchange community from which the above high‐GC fragment may have been acquired by *A. baumannii* (or by other ACB member) *ompA* by illegitimate recombination [56–58]. The presence of identical high‐GC stretches at similar gene positions in different *A. baumannii ompA* variants/subvariants (Fig. 2a) not only supports the notion that the further dissemination of this particular fragment among them proceeded by lateral gene transfer and fine‐scale homologous recombination [55], but also that it confers some fitness advantage(s) in the human‐associated niches in which *A. baumannii* and other ACB species thrive [1].

### Intra- and inter-species exchange of *ompA* variants

**Fig. 3.**
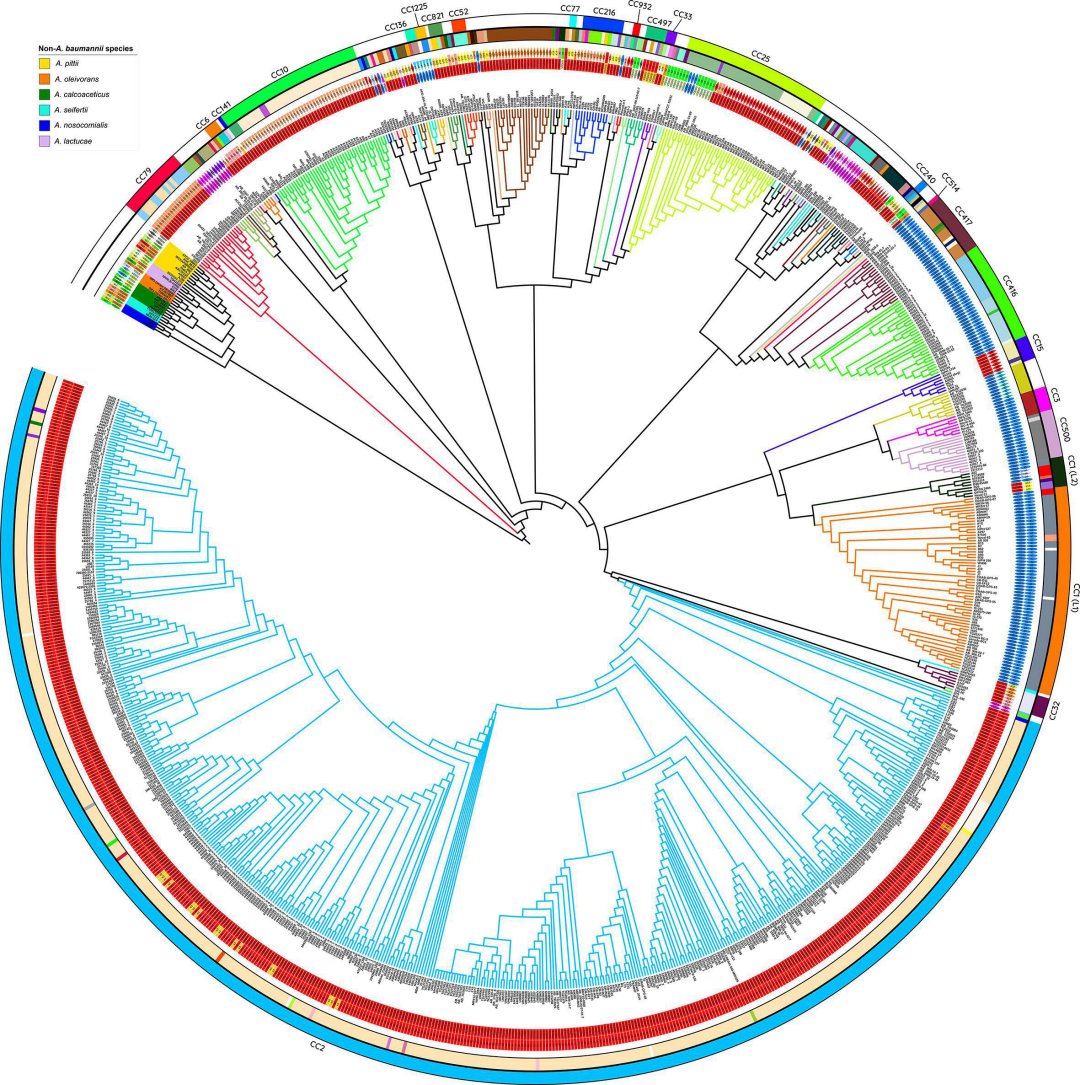
Evolutionary relatedness and *ompA* alleles of the *Acinetobacter* strains analysed in this work. An approximately ML core gene phylogeny was constructed using 474 core genes (present in ≥99 % of strains) with the non‐*baumannii* strain names coloured by species, as shown in the key. The tree is shown without scaling so that the relationships between strains can be seen, and is rooted on the branch separating the *A. baumannii* strains from the other *Acinetobacter* ACB species. The *ompA* gene alleles are indicated by the two rings of shapes surrounding the tree. The inner ring of shapes indicate the major V‐group that the *ompA* allele belongs to, and the outer ring indicates the specific gene allele. The two outermost circles indicate the MLST ST (inner circle) and CC (outer circle) according to the Pasteur MLST scheme, and the CCs are labelled (see Table S1 for details). Branches on the tree are coloured with respect to CC or ST.

We investigated the possibility of *ompA* exchange within different lineages of the *A *. *baumannii* population (intra‐species recombination) and also between different ACB species (inter‐species recombination). The overall observations are summarized in Fig. 3 (see also Tables S1 and S2 for further details). In agreement with other studies using different sets of core genes and phylogenetic strategies [1, 2, 9, 54, 60] the cluster formed by *A. baumannii* strains/isolates was clearly demarcated in the ML tree from those composed of its phylogenetically closely related ACB species including *A. nosocomialis *, *A. seifertii *, *A calcoaceticus *, *A. oleivorans *, *A. lactucae* and *A. pittii *, which were in turn well‐differentiated between them. The only exception was strain ANC3811, which has been assigned to *A *. *calcoaceticus* in the NCBI database (https://www.ncbi.nlm.nih.gov/genome/2267?genome_assembly_id=172286). In our ML analysis (Fig. 3) ANC3811 clustered with *A. oleivorans* strains, although as an outgroup of the other *A. oleivorans* strains employed. It is noteworthy in this context that ANC3811 contains an *ompA* V2(lct) allele similarly to *A. oleivorans* KCJK7897 and TUM15571 (Fig. 3), and that we could not detect V2(lct) genes among other *A. calcoaceticus* genome sequences deposited in the NCBI database (data not shown). Given the high similarity between *A. calcoaceticus* and *A. oleivorans* at the genome level [61], ANC3811 may thus well represent a taxonomic outlier close to both of these two species but not strictly belonging to either of them.

Several inferences could be derived from the analysis shown in Fig. 3. First, the *A. baumannii* strains/isolates comprising the major clonal complexes (as defined by the Institut Pasteur MLST scheme of population structure analysis, ref. 9): CC1, CC2 and CC3, as well as other CCs and STs of lower representation, formed well‐differentiated sub‐clusters in the ML tree of Fig. 3.These CCs are estimated to have evolved after 1960 from the clonal expansion each of a highly adaptive independent genotype, and they are generally thought to represent adaptations to specific microniches and be robust with respect to gene choice [4, 5, 8–10]. Second, *ompA* V1(lct) alleles were found widely distributed among the different branches of the *A. baumannii* cluster, with the exception of a few CC lineages (see below). This, added to the elevated frequency of V1(lct) alleles in the *A. baumannii* clinical population analysed (Table 1) and their presence already in the clinical strains ATCC 17978 and ATCC 19606 (see above) supports the notion that V1(lct) alleles may not only represent important determinants in the interaction of *A. baumannii* with the human host, but also may have even predated the founding genotypes of the different CCs. Third, the distribution of *ompA* variants between different *A. baumannii* sub‐clusters (Fig. 3, Table S1) revealed non‐random associations of particular variants (or variant alleles) and certain CCs, and which have been summarized in Table 3. For instance, 96 % of the 502 CC2 strains/isolates analysed displayed the *ompA* V1(lct)a1 allele (Table 3). From the two other V1(lct) alleles found among CC2 isolates (Fig. 3), V1(lct)a2 (two isolates) encodes an OmpA protein identical to V1(lct)a1, and V1(lct)a3 (16 isolates) encodes a protein differing by a single conservative amino acid substitution located in the periplasmic domain (Table S2 and Figure S3). These observations indicated a strong pressure for the preservation of the protein sequence codified by V1(lct)a1 (or, more precisely, of its exposed EL regions) during the global dissemination/expansion of CC2. In addition, V1(lct)a1 was found in two other *A. baumannii* strains/isolates branching separately from the CC2 cluster: AB_2007‐09‐110‐01‐7 and NIPH70 (Fig. 3). This additionally suggests that their acquisition by these two strains was mediated by recombinatorial *ompA* exchanges in which the donor probably belonged to CC2. It is also worth noting that in the CC2 subtree the isolates carrying V1(lct)a3 are interspaced among V1(lct)a1‐possessing isolates rather than grouped in a single cluster (Fig. 3), as it would be expected if they would have evolved clonally from a common ancestor. The observations above thus indicate not only the existence of exchanges of *ompA* genes between CC2 strains and other *A. baumannii* lineages, but also within CC2 as well. Fourth, the majority of the CC1 strains/isolates analysed (67 out of 73, 91.8 %) displayed V2(lct)a1 (Fig. 3, Tables 3 and S1). Moreover, CC1 strains/isolates belonging to the main (L1) sublineage of this global clone, estimated to have separated from the CC1 founding genotype early during the 1970s [10], all displayed V2(lct)a1. This indicates that V2(lct)a1 was strongly preserved during the global dissemination of this CC1 sub‐lineage. Three other CC1 strains, in this case assigned to the L2 sub‐lineage [9] (Fig. 3), showed V2(lct)a6 differing from V2(lct)a1 by one amino acid substitution at EL2 (Table S2). It follows that V2(lct), and in particular V2(lct)a1, represents the predominant *ompA* variant associated with CC1. Still, three CC1 strains/isolates also belonging to the L2 sub‐lineage of CC1 showed instead V1(lct)a14 (Fig. 3). This supports the idea of complete *ompA* variant replacements occurring very early during CC1 evolution, and which may have contributed to a better adaptation of this lineage to the human host or the nosocomial environment. Fifth, strong links could be observed for other *A. baumannii* clonal lineages with particular *ompA* alleles, as is the case of ST3 and its phylogenetically related CC500 lineage with V2(lct)a1; CC416 also with V2(lct)a1; ST406 with V2(lct)a2; ST499 with V1(lct)a30; CC10 with V1(lct)a27; CC79 with V1(lct)a5, CC15 with V1(lct)a8, ST49 with V4(lct)a1, ST52 with V1(lct)a14, etc. (Fig. 3, Table 3). The association of ST52 strains including ATCC 19606 (described in 1948) and more contemporary isolates with V1(lct)a14 (Table S1) merits comment, since it indicates that this allele accompanied this lineage since at least 10 years before the estimated emergence of the major epidemic *A. baumannii* clonal complexes [8, 9]. Sixth, other *A. baumannii* clonal lineages on the contrary seemed less selective in their choice of *ompA* variants, including CC25 encompassing V1(lct)a8‐, V1(lct)a5‐, V2(lct)a1‐ and V3(sct)a1‐carrying isolates; CC497 carrying V1(sct)a1, V1(lct)a1 and V5(sct)a1; ST16 carrying different V4(sct) alleles (Fig. 3, Table 3). Among these examples, ST32 contained isolates carrying different V1(lct) alleles but that translated to identical proteins, and the same situation occurred with CC240 and CC821 (Table 3). These latter cases thus represent examples of different *ompA* gene allele exchanges but in which identical OmpA proteins were maintained (Table 3). The overall observations above disclosed the existence of frequent assortative exchanges of *ompA* genes in the *A. baumannii* population.

**Table 3.** Associations detected between *A. baumannii* clonal lineages and *ompA* variants described in this work. For details see Fig. 3, Tables S1 and S2

Clonal lineage	Observations
CC2	Among 502 CC2 isolates (474 ST2, 27 SLV, 1 DLV), 484 (96.4 %) carried V1(lct)a1; 2 (0.4 %) V2(lct)a2; and 16 (3.2 %) V1(lct)a3. CC2 thus contained 99.6 % (484/486) of all V1(lct)a1‐carrying isolates found in the *A. baumannii* population analysed. Only V1(lct)a1 was found in two other isolates branching separately from the CC2 cluster: AB_2007‐09‐110‐01‐7 and NIPH70, providing evidences of V1(lct) exchanges between CC2 and other lineages.
CC1	Among 73 CC1 isolates (60 ST1, 12 SLV, 1 DLV), 67 (91.8 %) carried V2(lct)a1 (91.8 %); 3 V2(lct)a6 (4.1 %); and other 3 V1(lct)a14 (4.1 %). CC1 thus contained 45.6 % (67/147) of all V2(lct)a1‐carrying isolates found in the *A. baumannii* population analyzed. In addition, all isolates assigned to the main L1 sublineage of CC1 carried V2(lct)a1, the few detected V2(lct)a6‐ and V1(lct)a14‐containing isolates were restricted to the L2 sublineage. Evidences of exchanges of different variants and variant alleles during the evolution of this global clone.
ST3	All eight ST3 isolates carried V2(lct)a1. ST3 contained 5.4 % (8/147) of all V2(lct)a1‐carrying isolates found in the *A. baumannii* population analysed.
CC500	All 14 CC500 (13 ST500, 1 SLV) carried V2(lct)a1. CC500 contained 9.5 % of all V2(lct)a1‐carrying isolates found in the *A. baumannii* population. This CC lineage is phylogenetically close to ST3.
CC416	All 30 CC416 isolates (13 ST416, 17 SLV) carried V2(lct)a1. CC416 contained 20.4 % of all V2(lct)a1‐carrying isolates found in the *A. baumannii* population analysed.
CC417	All 19 CC417 isolates (14 ST417, 3 SLV, 2 DLV) carried V2(lct)a1. CC417 contained 12.9 % of all V2(lct)a1‐carrying isolates found in the *A. baumannii* population analysed.
CC79	All 20 CC79 isolates (9 ST79, 11 SLV) carried V1(lct)a5. CC79 thus contained 90.9 % (20/22) of all V1(lct)a5‐carrying isolates found in the *A. baumannii* population analysed.
CC10	All 48 CC10 strains (43 ST10, 5 SLV) carried V1(lct)a27. CC10 thus contained 98 % (48/49) of all V1(lct)a27‐carrying isolates found in the *A. baumannii* population analysed.
CC15	All seven CC15 isolates (six ST15, one SLV) carried V1(lct)a8. CC15 contained 16.3 % (7/43) of all V1(lct)a8‐carrying isolates found in the *A. baumannii* population.
ST406	All nine ST406 isolates carried V2(lct)a2. Thus, ST406 contained all V2(lct)a2‐carrying isolates found in the *A. baumannii* population.
ST499	All 20 ST499 isolates carried V1(lct)a30. Thus, ST499 contained all V1(lct)a30‐carrying isolates found in the *A. baumannii* population.
CC52	Composed by four ST52 isolates all carrying V1(lct)a14, isolated worldwide during a time period spanning 66 years: ATCC^T^19606 (USA, 1948), MSP4‐16 (India, 2010), GTC 03324 and GTC 03329 (Japan, 2014). CC52 includes another SLV isolate, AB_TG19617, carrying V1(lct)a6.
ST49	All four ST49 isolates carried V4(lct)a1 alleles. ST49 thus contained 44.4 % (4/9) of all V4(lct)a1 isolates found in the *A. baumannii* population.
CC25	The 47 CC25 isolates carried different *ompA* variants: 2 (ST25) carried V1(lct)a5, 31 (21 ST25, 10 SLV) carried V1(lct)a8, 4 (ST25) carried V2(lct)a1, and 10 (8 ST25, 1 SLV, 1 DLV) carried V3(sct)a1. Evidence of exchanges of different *ompA* variants and variant alleles.
ST16	The eight ST16 isolates analyzed carried different V4(lct) alleles: 5 carried V4(lct)a1 (55.5 % of all V4(lct)a1 alleles), 2 carried V4(lct)a4, and one carried V4(lct)a7. Evidence of different V4(lct) allele exchanges.
CC497	The six CC497 isolates carried different variants: Naval‐82 (SLV), TG27391 (SLV), 1 106 579 (SLV) and 83 444 (DLV) carried V1(sct)a1; A200 (SLV) carried V5(sct)a1, and AB_2007‐09‐110‐01‐7 (DLV) carried V1(lct)a1. Evidence of different *ompA* variant exchanges.
CC516	The five CC516 isolates carried different variants: AB405E4 (ST516) and CR‐ D1 (SLV) carried V1(lct)a32, A9 (DLV) carried V1(sct)a1, NIPH 80 (DLV) and 1 440 422 (DLV) carried V1(lct)a14. Evidence of different variant and variant allele exchanges.
CC33	The three CC33 isolates carried different variants: NIPH615 (DLV) and A219 (SLV) both carried V1(lct)a24, TG02017 (ST33) carried V1(sct)a2. Evidences of *ompA* variant exchanges.
ST32	ST32 encompassed six isolates carrying different V1(lct) alleles: OIFC032, OIFC087, and OIFC099 carried V1(lct)a12, while 781 407, 1 525 283 and A147 displayed V1(lct)a13. The two V1(lct) alleles translate to identical proteins: G52S replacement as compared to V1(lct)a1. Evidence of V1(lct) allele exchange while maintaining the same OmpA characteristics.
CC216	This CC encompassed ten isolates: WC‐A‐92 (DLV); A185, A181, A134 and A173, (all four ST216) bearing V1(lct)a11; AB405E4 (DLV) bearing V1(lct)a32; A9 (ST216) containing V1(sct)a1; NCIMB8209 (SLV) containing V1(lct)a32; NIPH80 (DLV) and 1 440 422 (SLV) carrying V1(lct)a14. Evidence of *ompA* variant exchanges.
CC240	CC240 encompassed two avian isolates carrying different V1(lct) alleles: 01D3‐2 (ST240) carried V1(lct)a10 while 86II/2C (DLV) carried V1(lct)a16. All alleles translate to identical proteins: G52S replacement as compared to V1(lct)a1. Evidence of V1(lct) allele replacement while maintaining the OmpA protein characteristics.
CC821	CC821 encompassed four isolates, including three avian isolates carrying V1(lct)a14: LoGest3‐1 (ST821), 151/C (SLV), and Ganse Ei‐1 (DLV); and the clinical isolate A166B (SLV) displaying V1(lct)a10. Both alleles translate to the same protein: G52S replacement as compared to V1(lct)a1. Evidence of V1(lct) allele replacement while maintaining the same OmpA protein characteristics.

Evidence for *ompA* exchanges between *A. baumannii* and non‐*baumannii* ACB species could also be inferred from the analysis shown in Fig. 3. For instance, V1(lct) alleles were detected among *A. pittii *, *A. lactucae* and *A. oleivorans* strains, V1(sct) alleles among *A. pittii *, *A *. *nosocomialis* and *A. seifertii* strains, V2(lct) alleles among *A. oleivorans* and *A. calcoaceticus* strains, V3(sct) alleles among *A. nosocomialis *, *A. calcoaceticus* and *A. pittii* strains, and V5(sct) alleles among *A. pittii* strains (Fig. 3). It is also noteworthy that V4(lct) and V4(sct) alleles were found only in *A. baumannii* but not among non‐*baumannii* strains and, conversely, that V3(lct) and V5(lct) alleles were found only among non‐*baumannii* strains (Table S2). The latter may reflect differential role(s) of these OmpA variants in the occupation of the various niches in which these ACB members thrive [1], although other factors could not be discarded at present (see below).

We also noted that the length of the OmpA signal peptide may vary between different ACB members, and even between different OmpA variants (Table S3). Thus, *A. baumannii* pre‐OmpAs, regardless of the variant considered, always showed identical signal peptides of 22 amino acid residues. Also, and in spite of the non‐*baumannii* ACB species analysed (* A *. *nosocomialis *, *A. lactucae *, *A. oleivorans *, *A. seifertii* or *A. pittii *), V1(lct) and V1(sct) pre‐OmpAs always showed signal peptides also of 22 amino acid residues (Table S3). On the other hand, and with the notorious exception of *A. nosocomialis* WC‐487 V3(sct), all other OmpA variants present in non‐*baumannii* species showed identical transit peptides of a length of 21 amino acid residues (Table S3). In *E. coli* OmpA, mutations in the signal peptide can significantly alter the rate of precursor synthesis with dramatic effects on protein export and assembly in the OM and consequent cell toxicity [62]. OmpA variants represent the most abundant proteins in the OM of *Acinetobacter* species [3–6], and it is thus possible that the lengths of their signal peptides are subject of strong selection to obtain the most efficient export and assembly at the lowest cell cost. This compromise may also be extended not only to the particular OmpA variant considered, but also on the *Acinetobacter* species in which it is produced. Although this situation could in principle hinder successful exchanges of *ompA* variants between ACB species, this seems not to represent a critical limitation as judged by the presence of V2 alleles in *A. baumannii* and also in *A. oleivorans* and *A. calcoaceticus *; V3(sct) alleles in *A. baumannii *, *A. nosocomialis *, *A. calcoaceticus* and *A. pittii *; and V5(sct) alleles in *A. baumannii* and also in *A. pittii* (Fig. 3, Table S2). These exchanges certainly required the preservation of the *ompA* signal peptide coding region originally present in the recipient organism, and provide further evidence of fine‐scale intragenic recombination operating at the level of the *Acinetobacter ompA* locus.

## Discussion

The study of *A. baumannii ompA* microevolution conducted here provides clues on the extent to which selection in the clinical habitat has shaped evolution of this OmpA/OprF family member. We detected five well‐defined polymorphic OmpA variants in the *A. baumannii* population studied, primarily of clinical origin, with most of the variation occurring within the four exposed EL regions located at the N‐terminal β‐barrel domain of these proteins, and another in their C‐terminal region (Fig. 2). Our analyses here indicated that recombination, both assortative and fine‐scale, appeared as a significant agent driving evolution of *A *. *baumannii* OmpA variants. We found signs of different fine‐scale intra‐genic recombination among variants, some encompassing the EL regions which appeared to have occurred during their early stages of divergence from a recent common ancestor. Other events, which appear more subtle and more recent, result in different C‐terminal sequences in their otherwise highly conserved C‐terminal periplasmic domains (Fig. 2, Table S3). The existence of two alternate C‐terminal ends differing in an Ala‐rich tract not only among different OmpA variants but also within a given variant, as in the case of V1 and V4 in *A. baumannii* (Fig. 2), strongly points to fine‐scale homologous recombination affecting the conserved *ompA* gene 3′‐region as the likely cause of this variability. Our analysis additionally suggested that the high‐GC DNA fragment encoding this Ala‐rich tract represents a relatively recent acquisition resulting from illegitimate recombination occurring in an *A. baumannii* (or a closely related ACB species) member adapting to human‐associated environments. It is worth noting that OmpA variants bearing this C‐terminal Ala‐rich tract are found in 98 % of all strains/isolates of the *A. baumannii* population analysed (Table 1). *Acinetobacter* members can incorporate into their genomes short fragments of DNA taken from different sources, including the soil microcosms, by homologous or non‐homologous recombination processes [56–58]. In fact, natural transformation with highly fragmented (≥20 bp) and even partially damaged DNA has been shown to be much more efficient than spontaneous mutation to generate adjacent nucleotide polymorphism in their genomes [58]. The OmpA periplasmic domain contributes to the stability of the cell envelope by interacting non‐covalently with the peptidoglycan [28, 41] (Fig. S1), and its deletion in *A. baumannii* ATCC 17978 [ST437; V1(lct)a19 *ompA*; Fig. 3] has been found to result in an increased cell susceptibility to different antimicrobials including cell-wall synthesis inhibitors [63]. Also, in the carbapenem‐resistant *A. baumannii* strain AB5075 [ST1; V2(lct)a1 *ompA*; Fig. 3] the OmpA periplasmic domain has been found to associate with different OM and periplasmic proteins including an acquired OXA‐23 β‐lactamase [37]. Ala residues in proteins are strong promoters of the formation of α‐helices with strong propensity to insert in lipid membranes and adopt higher‐order structures stabilized by lateral, helix–helix interactions [64]. The insertion of the hydrophobic Ala tract at the OmpA C‐terminal end may have resulted in an increased envelope stability towards the various cell-wall‐damaging substances used in the nosocomial environment [30, 63, 65], thus providing the driving force for its selection during adaptation of *A. baumannii* to this particular niche.

The different OmpA variants were unequally distributed among the *A. baumannii* population, with V1(lct) alleles being found in around 76 % of the strains/isolates analysed and V2 alleles in around 18 % of them (Table 1). We also found a strong pressure for the preservation of alleles of these two variants during the dissemination and expansion of a number of *A *. *baumannii* clonal lineages, including the main clonal complexes CC1, CC2 and CC3 (Fig. 3). Thus, V1(lct)a1 *ompA* was found primarily associated with CC2, while V2(lct)a1 was predominantly found among the main L1 lineage of CC1 and in CC3 (Fig. 3, Table 3). This suggests that the characteristics evolved by V1(lct)a1 OmpA confer significant fitness advantages to the strains that compose CC2, and the same applies for V2(lct)a1 OmpA in the case of CC2 and CC3. Strains composing other *A. baumannii* clonal lineages, in contrast, were apparently less selective in their use of *ompA* variants, as in the case of CC25 in which isolates carrying V1(lct)a5, V1(lct)a8, V2(lct)a1 and V3(sct)a1 were found (Fig. 3, Table 3). Even in this case, however, 33 out of the 47 CC25 strains (i.e., 70 %) carried V1(lct) alleles, with a great preponderance of V1(lct)a8 (Table 3).

Significant genetic diversity is a hallmark of *A. baumannii *, even between phylogenetically closely related strains [3, 4, 7, 10, 12, 13]. Whole‐genome sequencing studies of CC2 and CC1 strains have indicated that most of this genomic diversity is driven by recombination, with genetic material derived from different *A. baumannii* strains co‐existing in the environment [4, 10, 12, 13]. Many of these exchanges induced loss or swapping‐out of exposed structures potentially recognized by host defenses, such as the capsular polysaccharide, the outer core lipo‐oligosaccharide, iron capture components, efflux pumps, replacements of the OM protein CarO by variant alleles, etc. [4, 10–13]. It is assumed that this process contributes to immune evasion, facilitating long‐term patient colonization and spread of the *A. baumannii* epidemic clones [10–13]. *A. baumannii* OmpA has been found capable of eliciting immune responses including the production of anti‐OmpA antibodies both in infected patients [19, 38] and experimentally infected mice [16, 22]. It is then noteworthy that, in sharp contrast with the other surface‐exposed macromolecules mentioned above, particular *ompA* V1 and V2 alleles have been strongly preserved during the dissemination and expansion of the major *A *. *baumannii* global clones CC1 and CC2. This preservation supports the notion that these two OmpA variants play pivotal roles in *A. baumannii* pathogenicity [3–7, 20, 21, 31–36]. Their escape from host defenses could result, at least in part, from the recently disclosed shielding by capsule polysaccharide production during the course of *A. baumannii* infection [66]. The reasons for the success of CC strains as human pathogens are still poorly understood, and each CC appears in fact able to express a multiplicity of virulence factors in different combinations [3–6]. This has led to proposals that the virulence potential of strains composing the different CCs may reside on polymorphic differences in shared virulence genes and/or the combinatory effect of multiple genes [3–6]. OmpA is in fact a moonlighting protein implicated in a multitude of separate functions that include structural roles, slow porin for metabolites and antimicrobials, participation in virulence traits such as adhesion to extracellular matrix proteins, binding to host-cell receptors, biofilm formation and cytotoxicity [3–6, 19, 20, 29–36]. Thus, the functions of a particular OmpA variant in some epidemic *A. baumannii* lineages could be more related to the recognition of a specific host (or host tissue) and/or to the induction of cytotoxicity in a close interplay with other pathogenicity factors, and thus its exchange for another variant (or even a different allele) may be strongly selected against. In other *A. baumannii* CC lineages more prone to explore different niches, in contrast, OmpA may not be as highly demanded for pathogenicity and thus exchanges can be more frequently observed among its members. In any case, the observation made in this work that *ompA* variants, variant alleles, or even *ompA* gene sectors can be effectively exchanged by either assortative or fine‐scale recombination not only among different *A. baumannii* lineages but also between different ACB species represents a worrying scenario, providing that any innovation affecting host specificity or persistence may be rapidly disseminated among all components of this expanded community.

Finally, there has been interest in developing a vaccine against *A. baumannii* using OmpA as immunogen [16–22, 38]. However, the recombinatorial potential of *ompA* variants shown in this work may pose severe limitations to the effectiveness of a vaccine based solely on a single OmpA variant. It remains to be tested whether polyvalent OmpA vaccines generated through directed protein evolution [67] using the analysis provided in this work could prevent infections due to *A. baumannii* strains bearing different *ompA* variants. Further investigations involving functional studies of different OmpA variants/variant alleles may shed light upon the relative strengths of selection acting upon these OM proteins and help the development of new strategies for combating *A. baumannii* and other ACB infections.

## Data Bibliography

A complete list of the GenBank assembly accession numbers, or PubMLST id numbers, for all of the *Acinetobacter* genome sequences used in this study are given in Table S6 (available in the online version of this article). Accession numbers for other bacterial species are provided in Table S5. Accession numbers for specific contigs containing *ompA* alleles are given in Table S1, and accession numbers for non‐*A. baumannii Acinetobacter* genomes containing specific *ompA* alleles are given in Table S2.

## Supplementary Data

Supplementary material 1

Supplementary material 2
