# Supplementary material for: Microevolution in the major outer membrane protein OmpA of Acinetobacter baumannii
Source: Microb Genom. 2020 Jun 4;6(6):e000381. doi: 10.1099/mgen.0.000381 (PMC7371106; doi:10.1099/mgen.0.000381)
Supplement: Supplementary material 1 [file mgen-6-381-s001.pdf]

Figure S1: Protein sequence comparisons, predicted outer membrane topology, and relevant secondary structure elements of the different *A. baumannii* OmpA variants. Alignments were conducted using ClustalW (http://www.genome.jp/tools/clustalw/) and refined by visual inspection. The different OmpA variants described in *A. baumannii* are indicated in the left column, the numbers at the right indicate the position of the last aa residue for the corresponding variant in a particular row. The symbols below the alignments indicate identical (*) or conserved (:) aa residues at a given position, deletions are indicated in the sequences by hyphens (-). Topology predictions were done using PRED-TMBB (http://bioinformatics.biol.uoa.gr/PRED-TMBB/), N-terminal transit peptides are highlighted in yellow, periplasmic regions in green, transmembrane (TM) regions in red, and external loops (EL) in light blue. At the C-terminal periplasmic domain the $\alpha 2$ helix region (bearing the **N**X_2**LS**X_2**RA**X_2**V**X_2**L** conserved motif) and the $\alpha 3$ helix region, both forming part of the cavity interacting with the peptidoglycan common to OmpA-like domain proteins (see refs. 28 and 41), are indicated with "α" symbols above the alignments. The Arg residue located in $\alpha 2$ directly interacting with the diaminopimelate residue of the peptidoglycan is indicated by a closed arrowhead below the alignments. The $\alpha 3$ helix region encompasses the KTKEGRAMNRR signal found to be involved in the targeting of the OmpA from the ATCC 19606 strain to the nucleus of eukaryotic host cells (ref. 31). Note the 6 aa tract indel (AAAPAA, indicated in magenta) near the C-terminal region resulting in two alternate C-terminal ends of different lengths and hydrophobicity.

```
                     Transit peptide          TM1           EL1
V1(lct)a1      MKLSRIALATMLVAAPLAAANAGVTVTPLLLGYTFQDSQHNNGGKDGNLTNSPELQDDLF  60
V1(sct)a1      MKLSRIALATMLVAAPLAAANAGVTVTPLLLGYTFQDSQHNNGGKDGNLTNGPELQDDLF  60
V2(lct)a1      MKLSRIALATMLVAAPLAAANAGVTVTPLLLGYTFQDTQHNNGGKDGELTNGPELQDDLF  60
V3(sct)a1      MKLSRIALATMLVAAPLAAANAGVTVTPLLLGYTWQDSEHNN----NKLTDHAELQDDLF  56
V4(lct)a1      MKLSRIALATMLVAAPLAAANAGVTVTPLLLGYTFQDSEHNN----HKLTDSPELQDDLF  56
V4(sct)a1      MKLSRIALATMLVAAPLAAANAGVTVTPLLLGYTFQDSEHNN----HKLTDSPELQDDLF  56
V5(sct)a1      MKLSRIALATMLVAAPLAAANAGVTVTPLLLGYTFQDSQHNNGGKDGSLTNGPELQDDLF  60
               **********************************:**::***      **: :*******

                 TM2         TM3           EL2            TM4          TM5
V1(lct)a1      VGAALGIELTPWLGFEAEYNQVKGDVDGASA-GAEYKQKQINGNFYVTSDLITKNYDSKI 119
V1(sct)a1      VGAALGIELTPWLGFEAEYNQVKGDVDGASA-GAEYKQKQINGNFYVTSDLITKNYDSKI 119
V2(lct)a1      VGAALGIELTPWLGFEAEYNQVKGDVDGLAA-GAEYKQKQINGNFYVTSDLITKNYDSKI 119
V3(sct)a1      VGAGLGVELTPWLGFEAEYNQVKGDLDGTGVQGAEYKQKTIAGNFYATSDLITKNYDSKF 116
V4(lct)a1      VGAALGIELTPWLGFEAEYNQVKGDVD---TNYGEYKQKQINGNFYVTSDLITKNYDSKI 113
V4(sct)a1      VGAALGIELTPWLGFEAEYNQVKGDVD---PNYGEYKQKQINGNFYVTSDLITKNYDSKI 113
V5(sct)a1      VGAALGIELTPWLGFEAEYNQVKGDVD---TNYGEYKQKQINGNFYVTSDLITKNYDSKI 117
               *** **:******************:*       ***** * **** ************:

               TM5             EL3               TM6              TM7   EL4
V1(lct)a1      KPYVLLGAGHYKYDFDGVNRGTRGTSEEGTLGNAGVGAFWRLNDALSLRTEARATYNADE 179
V1(sct)a1      KPYVLLGAGHYKYDFDGVNRGTRGTSEEGTLGNAGVGAFWRLNDALSLRTEARATYNADE 179
V2(lct)a1      KPYVLLGAGHYKYEIPDL---SYHNDEEGTLGNAGVGAFWRLNDALSLRTEARGTYNFDE 176
```

```
V3(sct)a1         KPYVLLGAGQTKTEFDGI-----YEDKKDTIGNAGVGAFYRLNDALSLRTEARGTYDFDE 171
V4(lct)a1         KPYVLLGAGHYKYDFDDA-RLAYHDGEEGTLGNAGVGAFWRLNDALSLRTEARGTYNFDE 172
V4(sct)a1         KPYVLLGAGHYKYDFDDA-RLAYHDGEEGTLGNAGIGAFWRLNDALSLRTEARGTYNFDE 172
V5(sct)a1         KPYVLLGAGHYKYDFDDA-RLAYHDGEEGTLGNAGVGAFWRLNDALSLRTEARGTYNFDE 176
                  *********: * ::           :: *:****:***:************* **: **

                  EL4     TM8
V1(lct)a1         EFWNYTALAGLNVVLGGHLKPAAPVVEVAPVEPTPVAPQPQELTEDLNMELRVFFDTNKS 239
V1(sct)a1         EFWNYTALAGLNVVLGGHLKPAAPVVEVAPVEPTPVAPQPQELTEDLNMELRVFFDTNKS 239
V2(lct)a1         KFWNYTALAGLNVVLGGHLKPAAPVVEVAPVEPTPVAPQPQELTEDLNMELRVFFDTNKS 236
V3(sct)a1         KYWRYTALAGLNVVLGGHLKPAAPVVEVAPVEPTPVAPQPQELTEDLNMELRVFFDTNKS 231
V4(lct)a1         KFWNYTALAGLNVVLGGHLKPAAPVVEVAPVEPTPVAPQPQELTEDLNMELRVFFDTNKS 232
V4(sct)a1         KFWNYTALAGLNVVLGGHLKPAAPVVEVAPVEPTPVAPQPQELTEDLNMELRVFFDTNKS 232
V5(sct)a1         QFWNYTALAGLNVVLGGHLKPAAPVVEVAPVEPTPVAPQPQELTEDLNMELRVFFDTNKS 236
                  ::* ********************************************************

                                                                 α2
                                                     ααααααααααααααααααααααα
V1(lct)a1         NIKDQYKPEIAKVAEKLSEYPNATARIEGHTDNTGPRKLNERLSLARANSVKSALVNEYN 299
V1(sct)a1         NIKDQYKPEIAKVAEKLSEYPNATARIEGHTDNTGPRKLNERLSLARANSVKSALVNEYN 299
V2(lct)a1         NIKDQYKPEIAKVAEKLSEYPNATARIEGHTDNTGPRKLNERLSLARANSVKSALVNEYN 296
V3(sct)a1         NIKDQYKPEIAKVAEKLSEYPNATARIEGHTDNTGPRKLNERLSLARANSVKSALVNEYN 291
V4(lct)a1         NIKDQYKPEIAKVAEKLSEYPNATARIEGHTDNTGPRKLNERLSLARANSVKSALVNEYN 292
V4(sct)a1         NIKDQYKPEIAKVAEKLSEYPNATARIEGHTDNTGPRKLNERLSLARANSVKSALVNEYN 292
V5(sct)a1         NIKDQYKPEIAKVAEKLSEYPNATARIEGHTDNTGPRKLNERLSLARANSVKSALVNEYN 296
                  **********************************************▲*************

                                              α3
                                           αααααααα
V1(lct)a1         VDASRLSTQGFAWDQPIADNKTKEGRAMNRRVFATITGSRTVVVQPGQEAAAPAAAQ 356
V1(sct)a1         VDASRLSTQGFAWDQPIADNKTKEGRAMNRRVFATITGSRTVVVQPGQQ------AQ 350
V2(lct)a1         VDASRLSTQGFAWDQPIADNKTKEGRAMNRRVFATITGSRTVVVQPGQEAAAPAAAQ 353
V3(sct)a1         VDASRLSTQGFAWDQPIADNKTKEGRAMNRRVFATITGSRTVVVQPGQQ------AQ 342
V4(lct)a1         VDASRLSTQGFAWDQPIADNKTKEGRAMNRRVFATITGSRTVVVQPGQEAAAPAAAQ 349
V4(sct)a1         VDASRLSTQGFAWDQPIADNKTKEGRAMNRRVFATITGSRTVVVQPGQQ------AQ 343
V5(sct)a1         VDASRLSTQGFAWDQPIADNKTKEGRAMNRRVFATITGSRTVVVQPGQQ------AQ 347
                  ************************************************:      **
```

Figure S2: Nucleotide sequence comparisons of *A. baumannii ompA* variants. DNA alignments were done using ClustalW (http://www.genome.jp/tools/clustalw/) and refined by visual inspection on the basis of the protein alignments of Fig. S1. As in the previous Figure, the *ompA* variant genes are indicated at the left and the numbers at the right indicate the position of the last nt residue of the corresponding gene in a particular row. Uppercase letters denote the same nt in all sequences at a given position. Deletions are indicated by hyphens (-). The 18-nt indel and the C per G substitution near the TAA end codon resulting in variable OmpA C-terminal motifs are indicated in magenta. The nt sections coding for the N-terminal transit peptides, periplasmic regions, transmembrane (TM) regions, and external loops (EL) are highlighted as in Fig. S1 above. Evidence for recombination was found by using the RDP4 sofware (47) between V4 (major parent) and V3 (minor parent) at the EL1 region encompassing nt positions 121 to 141 of these sequences (4 programs out of 7, shadowed in light gray). RDP4 also indicated recombination (6 programs out of 7) between V4 and V5 at a gene region spanning nt positions 343 (V4)/355 (V5) to 512 (V4)/524 (V5), which encompasses the entire EL3 and part of EL4 (shadowed in gray).

```
                                  Transit peptide coding region
V1(lct)a1    ATGAAATTGAGTCGTATTGCACTTGCTACTATGCTTGTTGCTGCTCCATTAGCTGCTGCT   60
V1(sct)a1    ATGAAATTGAGTCGTATTGCACTTGCTACTATGCTTGTTGCTGCTCCATTAGCTGCTGCT   60
V2(lct)a1    ATGAAATTGAGTCGTATTGCACTTGCTACTATGCTTGTTGCTGCTCCATTAGCTGCTGCT   60
V3(sct)a1    ATGAAATTGAGTCGTATTGCACTTGCTACTATGCTTGTTGCTGCTCCATTAGCTGCTGCT   60
V4(lct)a1    ATGAAATTGAGTCGTATTGCACTTGCTACTATGCTTGTTGCTGCTCCATTAGCTGCTGCT   60
V4(sct)a1    ATGAAATTGAGTCGTATTGCACTTGCTACTATGCTTGTTGCTGCTCCATTAGCTGCTGCT   60
V5(sct)a1    ATGAAATTGAGTCGTATTGCACTTGCTACTATGCTTGTTGCTGCTCCATTAGCTGCTGCT   60

                                              TM1                  EL1
V1(lct)a1    AATGCTGGCGTAACAGTTACTCCATTAcTGCTTGGTTAcACtTtcCAAGAcagccAaCAc  120
V1(sct)a1    AATGCTGGCGTAACAGTTACTCCATTATTGCTTGGTTAcACtTtcCAAGAcagccAaCAc  120
V2(lct)a1    AATGCTGGCGTAACAGTTACTCCATTATTGCTTGGTTAcACtTtcCAAGAcactcAaCAc  120
V3(sct)a1    AATGCTGGCGTAACAGTTACTCCATTATTGCTTGGTTAtACtTggCAAGAcagcgAgCAc  120
V4(lct)a1    AATGCTGGCGTAACAGTTACTCCATTATTGCTTGGTTAcACaTtcCAAGAttctgAaCAt  120
V4(sct)a1    AATGCTGGCGTAACAGTTACTCCATTATTGCTTGGTTAcACaTtcCAAGAttctgAaCAt  120
V5(sct)a1    AATGCTGGCGTAACAGTTACTCCATTATTGCTTGGTTAcACtTtcCAAGAcagccAaCAc  120

                                  EL1
V1(lct)a1    AACAAtggcggtaaagatggtaacTTaACtaAttcacCtGAgtTaCAAGAcGATTTATTC  180
V1(sct)a1    AACAAtggcggtaaagatggtaacTTaACtaAtgcacCtGAgtTaCAAGAcGATTTATTC  180
V2(lct)a1    AACAAtggcggtaaagatggcgagTTaACtaAcggacCtGAatTaCAAGAcGATTTATTC  180
V3(sct)a1    AACAAc------------aataaaTTaACtgAtcatgCtGAatTgCAAGAtGATTTATTt  168
V4(lct)a1    AACAAc------------cataaaTTaACtgAtagccCaGAgcTaCAAGAtGATTTATTt  168
V4(sct)a1    AACAAc------------cataaaTTaACtgAtagccCaGAgcTaCAAGAtGATTTATTt  168
V5(sct)a1    AACAAtggcggtaaagatggtagcTTgACaaAtggtcCtGAgtTaCAAGAcGATTTATTC  180

                 TM2                                   TM3
V1(lct)a1    GTTGGtGCaGcTCTgGGTaTcGAGTTAACtCCTTGGTTAGGTTTcGAAGCTGAATATAAc  240
V1(sct)a1    GTTGGcGCaGcTCTgGGTaTcGAGTTAACtCCTTGGTTAGGTTTcGAAGCTGAATATAAc  240
```

```
V2(lct)a1     GTTGGtGCaGcTCTTGGTaTcGAGTTAACaCCTTGGTTAGGTTTcGAAGCTGAATATAAc   240
V3(sct)a1     GTTGGtGCtGgTCTgGGTgTtGAGTTAACaCCTTGGTTAGGTTTtGAAGCTGAATATAAt   228
V4(lct)a1     GTTGGtGCaGcTCTtGGTaTcGAGTTAACaCCTTGGTTAGGTTTcGAAGCTGAATATAAc   228
V4(sct)a1     GTTGGtGCaGcTCTtGGTaTcGAGTTAACaCCTTGGTTAGGTTTcGAAGCTGAATATAAc   228
V5(sct)a1     GTTGGtGCaGcTCTtGGTaTcGAGTTAACaCCTTGGTTAGGTTTtGAAGCTGAATATAAc   240

                               EL2
V1(lct)a1     CAAGTtAAAGGcGAcgTaGAcggcgcttctgct---ggtGcTGAaTAtAAaCAaAAacaa   297
V1(sct)a1     CAAGTtAAAGGcGAcgTaGAcggcgcttctgct---ggtGcTGAaTAtAAaCAaAAacaa   297
V2(lct)a1     CAAGTtAAAGGtGAtgTaGAcggtcttgcagct---ggcGcTGAaTAcAAgCAaAAacaa   297
V3(sct)a1     CAAGTaAAAGGtGAtcTtGAtggtactggcgttcaaggcGcTGAgTAcAAaCAgAAgact   288
V4(lct)a1     CAAGTtAAAGGtGAtgTaGAc---------acgaactatGgTGAaTAtAAaCAgAAacaa   279
V4(sct)a1     CAAGTtAAAGGtGAtgTaGAt---------ccaaactacGgTGAaTAtAAgCAgAAacaa   279
V5(sct)a1     CAAGTtAAAGGtGAtgTaGAc---------acgaactatGgTGAaTAcAAaCAaAAacaa   291

                        TM4                                  TM5
V1(lct)a1     ATcaacGGTAACTTCTATGttACTTCTGATTTAATtACTAAAAACTAcGAcAGCAAAaTC   357
V1(sct)a1     ATcaacGGTAACTTCTATGttACTTCTGATTTAATtACTAAAAACTAcGAcAGCAAAaTC   357
V2(lct)a1     ATcaacGGTAACTTCTATGttACTTCTGATTTAATcACTAAAAACTAtGAcAGCAAAaTC   357
V3(sct)a1     ATtgctGGTAACTTCTATGcaACTTCTGATTTAATcACTAAAAACTAtGAcAGCAAAtTt   348
V4(lct)a1     ATcaacGGTAACTTCTATGttACTTCTGATTTAATtACTAAAAACTAcGAtAGCAAAaTC   339
V4(sct)a1     ATcaatGGTAACTTCTATGttACTTCTGATTTAATtACTAAAAACTAcGAtAGCAAAaTC   339
V5(sct)a1     ATcaaCGGTAACTTCTATGttACTTCTGATTTAATtACTAAAAACTAtGAcAGCAAAaTC   351

                                                       EL3
V1(lct)a1     AAgCCgTAcGTATTaTTAGGTGCtGGTCActatAAAtatGAcTTGaTGgcgtaaatcgt   417
V1(sct)a1     AAgCCgTAcGTATTaTTAGGTGCtGGTCActatAAAtacGAcTTGaTGgcgtaaaccgt   417
V2(lct)a1     AAgCCaTAcGTATTgTTAGGTGCtGGTCActacAAAtacGAgaTTccTGacctt------   411
V3(sct)a1     AAgCCaTAcGTATTgTTAGGTGCgGGTCAaacTAAAactGAgtTTgaTGgtatc------   402
V4(lct)a1     AAaCCtTAcGTATTgTTAGGTGCaGGTCAttatAAAtatGAttTTgaTGatgct---cgt   396
V4(sct)a1     AAaCCtTAcGTATTgTTAGGTGCaGGTCAttatAAAtatGAttTTgaTGatgct---cgt   396
V5(sct)a1     AAgCCtTAtGTATTgTTAGGTGCaGGTCAttatAAAtatGAttTTgaTGatgct---cgt   408

                                                          TM6
V1(lct)a1     ggtacacgtggtacttctgAagAAGgTACTtTaGGTAAcGCTGGTgTtGGTGCTTTCTgg   477
V1(sct)a1     ggtaCacgtggtacttctgAagAAGgTACTtTaGGTAAcGCTGGTgTtGGTGCTTTCTgg   477
V2(lct)a1     ---tcttatcacaacgatgAagAAGgTACTtTaGGTAAtGCTGGTgTtGGTGCTTTCTgg   468
V3(sct)a1     ---------tatgaagacaAgaAAGaTACTaTcGGTAAtGCcGGTgTaGGTGCTTTCTat   453
V4(lct)a1     ttagcttaccatgatggtgAagAAGgTACTtTaGGTAAcGCTGGTgTtGGTGCTTTCTgg   456
V4(sct)a1     ttagcttaccatgatggtgAagAAGgTACTtTaGGTAAcGCTGGTaTtGGTGCTTTCTgg   456
V5(sct)a1     ttagcttaccatgatggtgAagAAGgTACTtTaGGTAAtGCTGGTgTtGGTGCTTTCTgg   468

                                   TM7                      EL4
V1(lct)a1     CGCTTaAAcGAcGCTtTaTCTCTTCGTACtGAAGCTCGtGcTACtTATaAtgcTGAtGAA   537
V1(sct)a1     CGCTTaAAcGAcGCTtTaTCTCTTCGTACtGAAGCTCGtGcTACtTATaAtgcTGAtGAA   537
V2(lct)a1     CGCTTaAAcGAtGCTcTaTCTCTTCGTACaGAAGCTCGtGgTACtTATaActtTGAcGAA   528
V3(sct)a1     CGCTTgAAcGAtGCTTTgTCTCTTCGTACaGAAGCTCGcGgTACgTATgAtttTGAtGAA   513
V4(lct)a1     CGCTTaAAtGAtGCTtTaTCTCTTCGTACaGAAGCTCGtGgTACtTATaActtTGAcGAA   516
V4(sct)a1     CGCTTaAAcGAtGCTtTaTCTCTTCGTACaGAAGCTCGtGgTACtTATaActtTGAcGAA   516
V5(sct)a1     CGCTTaAAtGAcGCTtTaTCTCTTCGTACaGAAGCgCGtGgTACtTATaActtTGAtGAA   528

                                  TM8
V1(lct)a1     gAgTtCTGGaaCTAtACaGCtCTTGCTGGCTTAAACGTAGTTCTTGGTGGTCACTTGAAG   597
V1(sct)a1     gAgTtCTGGaaCTAtACaGCtCTTGCTGGCTTAAACGTAGTTCTTGGTGGTCACTTGAAG   597
V2(lct)a1     aAaTtCTGGaaCTAtACaGCtCTTGCTGGCTTAAACGTAGTTCTTGGTGGTCACTTGAAG   588
V3(sct)a1     aAaTaCTGGcgCTAcACtGCtCTTGCTGGCTTAAACGTAGTTCTaGGTGGTCACTTGAAG   573
V4(lct)a1     aAaTtCTGGaaCTAtACaGCtCTTGCTGGCTTAAACGTAGTTCTTGGTGGTCACTTGAAG   576
V4(sct)a1     aAaTtCTGGaaCTAtACaGCaCTTGCTGGCTTAAACGTAGTTCTTGGTGGTCACTTGAAG   576
V5(sct)a1     cAaTtCTGGaaCTAtACaGCtCTTGCTGGCTTAAACGTAGTTCTTGGTGGTCACTTGAAG   588

V1(lct)a1     CCTGCTGCTCCTGTAGTAGAAGTTGCTCCAGTTGAACCAACTCCAGTTGCTCCACAACCA   657
V1(sct)a1     CCTGCTGCTCCTGTAGTAGAAGTTGCTCCAGTTGAACCAACTCCAGTTGCTCCACAACCA   657
V2(lct)a1     CCTGCTGCTCCTGTAGTAGAAGTTGCTCCAGTTGAACCAACTCCAGTTGCTCCACAACCA   648
V3(sct)a1     CCTGCTGCTCCTGTAGTAGAAGTTGCTCCAGTTGAACCAACTCCAGTTGCTCCACAACCA   633
V4(lct)a1     CCTGCTGCTCCTGTAGTAGAAGTTGCTCCAGTTGAACCAACTCCAGTTGCTCCACAACCA   636
V4(sct)a1     CCTGCTGCTCCTGTAGTAGAAGTTGCTCCAGTTGAACCAACTCCAGTTGCTCCACAACCA   636
V5(sct)a1     CCTGCTGCTCCTGTAGTAGAAGTTGCTCCAGTTGAACCAACTCCAGTTGCTCCACAACCA   648

V1(lct)a1     CAAGAGTTAACTGAAGACCTTAACATGGAACTTCGTGTgTTCTTTGATACTAACAAATCA   717
V1(sct)a1     CAAGAGTTAACTGAAGACCTTAACATGGAACTTCGTGTgTTCTTTGATACTAACAAATCA   708
```

```
V2(lct)a1      CAAGAGTTAACTGAAGACCTTAACATGGAACTTCGTGTgTTCTTTGATACTAACAAATCA  693
V3(sct)a1      CAAGAGTTAACTGAAGACCTTAACATGGAACTTCGTGTgTTCTTTGATACTAACAAATCA  717
V4(lct)a1      CAAGAGTTAACTGAAGACCTTAACATGGAACTTCGTGTaTTCTTTGATACTAACAAATCA  696
V4(sct)a1      CAAGAGTTAACTGAAGACCTTAACATGGAACTTCGTGTgTTCTTTGATACTAACAAATCA  708
V5(sct)a1      CAAGAGTTAACTGAAGACCTTAACATGGAACTTCGTGTgTTCTTTGATACTAACAAATCA  696

V1(lct)a1      AACATCAAAGACCAaTACAAGCCAGAAATcGCTAAAGTTGCTGAAAAATTATCTGAATAC  777
V1(sct)a1      AACATCAAAGACCAaTACAAGCCAGAAATcGCTAAAGTTGCTGAAAAATTATCTGAATAC  777
V2(lct)a1      AACATCAAAGACCAaTACAAGCCAGAAATcGCTAAAGTTGCTGAAAAATTATCTGAATAC  768
V3(sct)a1      AACATCAAAGACCAgTACAAGCCAGAAATcGCTAAAGTTGCTGAAAAATTATCTGAATAC  753
V4(lct)a1      AACATCAAAGACCAaTACAAGCCAGAAATtGCTAAAGTTGCTGAAAAATTATCTGAATAC  756
V4(sct)a1      AACATCAAAGACCAaTACAAGCCAGAAATcGCTAAAGTTGCTGAAAAATTATCTGAATAC  756
V5(sct)a1      AACATCAAAGACCAaTACAAGCCAGAAATtGCTAAAGTTGCTGAAAAATTATCTGAATAC  768

V1(lct)a1      CCTAACGCTACTGCACGTATCGAAGGTCAcACAGATAACACTGGTCCACGTAAGTTGAAC  837
V1(sct)a1      CCTAACGCTACTGCACGTATCGAAGGTCAcACAGATAACACTGGTCCACGTAAGTTGAAC  837
V2(lct)a1      CCTAACGCTACTGCACGTATCGAAGGTCAcACAGATAACACTGGTCCACGTAAGTTGAAC  828
V3(sct)a1      CCTAACGCTACTGCACGTATCGAAGGTCAcACAGATAACACTGGTCCACGTAAGTTaAAC  813
V4(lct)a1      CCTAACGCTACTGCACGTATCGAAGGTCAcACAGATAACACTGGTCCACGTAAGTTGAAC  816
V4(sct)a1      CCTAACGCTACTGCACGTATCGAAGGTCAtACAGATAACACTGGTCCACGTAAGTTGAAC  816
V5(sct)a1      CCTAACGCTACTGCACGTATCGAAGGTCAcACAGATAACACTGGTCCACGTAAGTTGAAC  828

V1(lct)a1      GAACGTTTATCTTTAGCTCGTGCTAACTCTGTTAAATCAGCTCTTGTAAACGAATAcAAC  897
V1(sct)a1      GAACGTTTATCTTTAGCTCGTGCTAACTCTGTTAAATCAGCTCTTGTAAACGAATAtAAC  897
V2(lct)a1      GAACGTTTATCTTTAGCTCGTGCTAACTCTGTTAAATCAGCTCTTGTAAACGAATAcAAC  888
V3(sct)a1      GAACGTTTATCTTTAGCTCGTGCTAACTCTGTTAAATCAGCTCTTGTAAACGAATAcAAC  873
V4(lct)a1      GAACGTTTATCTTTAGCTCGTGCTAACTCTGTTAAATCAGCTCTTGTAAACGAATAcAAC  876
V4(sct)a1      GAACGTTTATCTTTAGCTCGTGCTAACTCTGTTAAATCAGCTCTTGTAAACGAATAtAAC  876
V5(sct)a1      GAACGTTTATCTTTAGCTCGTGCTAACTCTGTTAAATCAGCTCTTGTAAACGAATAtAAC  888

V1(lct)a1      GTTGACGCTTCTCGTTTGTCTACTCAAGGTTTCGCTTGGGATCAACCGATTGCTGACAAC  957
V1(sct)a1      GTTGACGCTTCTCGTTTGTCTACTCAAGGTTTCGCTTGGGATCAACCGATTGCTGACAAC  957
V2(lct)a1      GTTGACGCTTCTCGTTTGTCTACTCAAGGTTTCGCTTGGGATCAACCGATTGCTGACAAC  948
V3(sct)a1      GTTGACGCTTCTCGTTTGTCTACTCAAGGTTTCGCTTGGGATCAACCGATTGCTGACAAC  933
V4(lct)a1      GTTGACGCTTCTCGTTTGTCTACTCAAGGTTTCGCTTGGGATCAACCGATTGCTGACAAC  936
V4(sct)a1      GTTGACGCTTCTCGTTTGTCTACTCAAGGTTTCGCTTGGGATCAACCGATTGCTGACAAC  936
V5(sct)a1      GTTGACGCTTCTCGTTTGTCTACTCAAGGTTTCGCTTGGGATCAACCGATTGCTGACAAC  948

V1(lct)a1      AAAACTAAAGAAGGTCGTGCTATGAACCGTCGTGTATTCGCGACAATCACTGGTAGCCGT 1017
V1(sct)a1      AAAACTAAAGAAGGTCGTGCTATGAACCGTCGTGTATTCGCGACAATCACTGGTAGCCGT 1017
V2(lct)a1      AAAACTAAAGAAGGTCGTGCTATGAACCGTCGTGTATTCGCGACAATCACTGGTAGCCGT 1008
V3(sct)a1      AAAACTAAAGAAGGTCGTGCTATGAACCGTCGTGTATTCGCGACAATCACTGGTAGCCGT  993
V4(lct)a1      AAAACTAAAGAAGGTCGTGCTATGAACCGTCGTGTATTCGCGACAATCACTGGTAGCCGT  996
V4(sct)a1      AAAACTAAAGAAGGTCGTGCTATGAACCGTCGTGTATTCGCGACAATCACTGGTAGCCGT  996
V5(sct)a1      AAAACTAAAGAAGGTCGTGCTATGAACCGTCGTGTATTCGCGACAATCACTGGTAGCCGT 1008

V1(lct)a1      ACTGTAGTAGTTCAACCTGGTCAAgAAgcggcagctcctgcagcaGCTCAATAA 1071
V1(sct)a1      ACTGTAGTAGTTCAACCTGGTCAAcAA------------------GCTCAATAA 1053
V2(lct)a1      ACTGTAGTAGTTCAACCTGGTCAAgAAgcggcagctcctgcagcaGCTCAATAA 1062
V3(sct)a1      ACTGTAGTAGTTCAACCTGGTCAAcAA------------------GCTCAATAA 1029
V4(lct)a1      ACTGTAGTAGTTCAACCTGGTCAAgAAgcggcagctcctgcagcaGCTCAATAA 1050
V4(sct)a1      ACTGTAGTAGTTCAACCTGGTCAAcAA------------------GCTCAATAA 1032
V5(sct)a1      ACTGTAGTAGTTCAACCTGGTCAAcAA------------------GCTCAATAA 1044
```

Figure S3. Synonymous and non-synonymous mutations among *Acinetobacter ompA* V1(lct) alleles. **A.** Comparisons of the total *ompA* V1(lct) nucleotide sequences described in this work in *A. baumannii* and non-*baumannii* strains/isolates. The DNA alignments of the indicated V1(lct) *ompA* alleles [32 from *A. baumannii*, 2 from *A. pittii* (strains XJ88 and 2012N08-034, respectively), 1 from *A. lactucae* (strain CI78), 1 from *A. oleivorans* (strain DR1, subspecies DE0008) were done using ClustalW (http://www.genome.jp/tools/clustalw/) using 1,071 nucleotide positions encompassing 356 codons covering the whole gene sequences. *A. baumannii ompA* alleles displaying the same DNA sequence in a given row are indicated at the left, with the numbers at the right indicating the position of the last nucleotide residue in this row. Nucleotide substitutions [as compared to V1(lct)a1] for a particular allele or group of alleles are indicated in bold red in the alignments, with the amino acid residue encoded by the triplet in which the mutations were detected (Aa involved) added below the alignments. It is also indicated in each case whether the type of nucleotide substitution represented a transition (t) or a transversion (T) event, and whether it resulted in a synonymous (Sy) or a non-synonymous (nSy) change. OM topology predictions for the encoded proteins are also indicated in the upper row, with the externally exposed (EL) coding regions highlighted in light blue and the non-exposed coding regions indicated in red (transmembrane) or green (periplasm). The amino acid substitutions unique to V1(lct)a1: Ser52 and Thr144, are highlighted in magenta. See legend to Figure S2 and Table S2 for further details. **B.** Intra- and inter-species amino acid substitutions and possible concomitant effects. The properties of the amino acids and whether the change is neutral, favored, or disfavored in the case of membrane proteins were from (M.J. Betts, R.B. Russell. Amino acid properties and consequences of substitutions. In Bioinformatics for Geneticists, M.R. Barnes, I.C. Gray eds, Wiley, 2003; http://www.russelllab.org/aas/). **C.** Summary of synonymous and non-synonymous substitutions at polymorphic sites detected between V1(lct) *ompA* alleles in the *Acinetobacter* population analyzed.

A)

```
a1-a32          ATGAAATTGAGTCGTATTGCACTTGCTACTATGCTTGTTGCTGCTCCATTAGCTGCTGCT   60
ap1             ATGAAATTGAGTCGTATTGCACTTGCTACTATGCTTGTTGCTGCTCCATTAGCTGCTGCT
ap2             ATGAAATTGAGTCGTATTGCACTTGCTACTATGCTTGTTGCTGCTCCATTAGCTGCTGCT
al1             ATGAAATTGAGTCGTATTGCACTTGCTACTATGCTTGTTGCTGCTCCATTAGCTGCTGCT
ao1             ATGAAATTGAGTCGTATTGCACTTGCTACTATGCTTGTTGCTGCTCCATTAGCTGCTGCT

                                             TM1
a1-a5           AATGCTGGCGTAACAGTTACTCCATTACTGCTTGGTTACACTTTCCAAGACAGCCAACAC  120
                                           Leu
a6-a32          AATGCTGGCGTAACAGTTACTCCATTATTGCTTGGTTACACTTTCCAAGACAGCCAACAC
Aa involved                                Leu
Type of change                             t
Syn or non-Syn                             Sy
ap1             AATGCTGGCGTAACAGTTACTCCATTATTGCTTGGTTACACTTTCCAAGACAGCCAACAC
ap2             AATGCTGGCGTAACAGTTACTCCATTATTGCTTGGTTACACTTTCCAAGACAGCCAACAC
al1             AATGCTGGCGTAACAGTTACTCCATTATTGCTTGGTTACACTTTCCAAGACAGCCAACAC
ao1             AATGCTGGCGTAACAGTTACTCCATTATTGCTTGGTTACACTTTCCAAGACAGCCAACAC
Aa involved                                Leu
Type of change                             t
Syn or non-Syn                             Sy
```

```
                                  EL1
a1-a7           AACAATGGCGGTAAAGATGGTAACTTAACTAATTCACCTGAGTTACAAGACGATTTATTC  180
Aa involved     AsnAsnGly            AsnLeu   AsnSer
a8,a21-a26      AACAATGGCGGTAAAGATGGTAACTTAACTAATGCACCTGAGTTACAAGACGATTTATTC
Aa involved                          AsnLeu   AsnAla
a9-a19,a27-30   AACAATGGCGGTAAAGATGGTAACTTAACTAACGGTCCTGAGTTACAAGACGATTTATTC
a15             AACAATGGCGGTAAAGATGGTAATTTAACTAACGGTCCTGAGTTACAAGACGATTTATTC
a31,a32         AACAATGGCGGTAAAGATGGTAACTTAACTAACGGACCTGAGTTACAAGACGATTTATTC
ap1             AACAATGGCGGTAAAGATGGTAACTTAACTAACGGACCTGAGTTACAAGACGATTTATTC
ap2             AACAATGGCGGTAAAGATGGTAACTTAACTAACGGACCTGAGTTACAAGACGATTTATTC
al1             AACAATGGTGGTAAAGATGGTAACTTAACTAACGGTCCTGAGTTACAAGACGATTTATTC
ao1             AATAATGGCGGTAAAGATGGTAACCTAACTAACGGTCCTGAGTTACAAGACGATTTATTC
Aa involved     AsnAsnGly            AsnLeu   AsnGly
Type of change    t     t              tt       tTTT
Syn or non-Syn   Sy     Sy           Sy Sy    Sy nSy

                       TM2                    IL1            TM3
a1-a7           GTTGGTGCAGCTCTTGGTATCGAGTTAACTCCATGGTTAGGTTTCGAAGCTGAATATAAC  240
a8, a20-26      GTTGGCGCAGCTCTTGGTATCGAGTTAACACCATGGTTAGGTTTCGAAGCTGAATATAAC
a9-a19, a27-a30 GTTGGCGCAGCTCTTGGTATCGAGTTAACTCCATGGTTAGGTTTCGAAGCTGAATATAAC
a31, a32        GTTGGTGCAGCTCTTGGTATCGAGTTAACACCATGGTTAGGTTTCGAAGCTGAATATAAC
ap1             GTTGGTGCAGCTCTTGGTATCGAGTTAACACCATGGTTAGGTTTCGAAGCTGAATATAAC
ap2             GTTGGTGCAGCTCTTGGTATCGAGTTAACACCATGGTTAGGTTTCGAAGCTGAATATAAC
al1             GTTGGTGCAGCTCTTGGTATCGAGTTAACACCATGGTTAGGTTTCGAAGCTGAATATAAC
ao1             GTTGGTGCAGCTCTTGGTATCGAGTTAACACCATGGTTAGGTTTCGAAGCTGAATATAAC
Aa involved        Gly                       Thr
Type of change       t                         T
Syn or non-Syn      Sy                        Sy

                               EL2
a1-6, a8-15,
a17-20, a27,28  CAAGTTAAAGGCGACGTAGACGGCGCTTCTGCTGGTGCTGAATATAAACAAAAACAAATC  300
a7, a16, a21-26 CAAGTTAAAGGTGACGTAGACGGCGCTTCTGCTGGTGCTGAATATAAACAAAAACAAATC
Aa involved              Gly         GlyAlaSerAlaGly
a31, a32        CAAGTTAAAGGCGACGTAGACGGCGCTGCTGCTGGTGCTGAATATAAACAAAAACAAATC
Aa involved                          GlyAlaAla
a29, a30        CAAGTTAAAGGCGACGTAGACGGTCCTGTTGCTGGTGCTGAATATAAACAAAAACAAATC
Aa involved                          GlyProVal
Type of change             t           tT  Tt
Syn or non-syn            Sy         Sy nSy nSy
ap1             CAAGTTAAAGGCGACGTAGACGGCGCTGCTGCTGGTGCTGAATATAAACAAAAACAAATC
ap2             CAAGTTAAAGGCGACGTAGACGGCGCTGCTGCTGGTGCTGAATATAAACAAAAACAAATC
al1             CAAGTTAAAGGCGACGTAGACGGCGCTGCTGCTGGTGCTGAATATAAACAAAAACAAATC
ao1             CAAGTTAAAGGTGACGTAGACGGTGCTGCTGCTGGCGCTGAATATAAACAAAAACAAATC
Aa involved              Gly         GlyAlaAla   Gly
Type of change             t           t   T       t
Syn or non-syn            Sy         Sy    nSy    Sy

                       TM4
a1-16, a18-30   AACGGTAACTTCTATGTTACTTCTGATTTAATTACTAAAAACTACGACAGCAAAATCAAG  360
a17             AATGGTAACTTCTATGTTACTTCTGATTTAATTACTAAAAACTACGACAGCAAAATCAAG
a31, a32        AACGGTAACTTCTATGTTACTTCTGATTTAATCACTAAAAACTACGACAGCAAAATCAAG
Aa involved     Asn                           Ile                     Ile
Type of change    t                             t
Syn or non-syn  Sy                             Sy
ap1             AACGGTAACTTCTATGTTACTTCTGATTTAATCACTAAAAACTACGACAGCAAAATCAAG
ap2             AATGGTAACTTCTATGTTACTTCTGATTTAATCACTAAAAACTACGACAGCAAAATCAAG
al1             AACGGTAACTTCTATGTTACTTCTGATTTAATCACTAAAAACTACGACAGCAAAATCAAG
ao1             AACGGTAACTTCTATGTTACTTCTGATTTAATCACTAAAAACTACGACAGCAAAATTAAG
Aa involved     Asn                           Ile                     Ile
Type of change    t                             t                       t
Syn or non-syn  Sy                             Sy                      Sy
```

```
                                                           EL3
a1-a4           CCGTACGTATTATTAGGTGCTGGTCACTATAAATATGACTTTGATGGCGTAAATCGTGGT   420
a5-10,a12-19    ProTyr   Leu               TyrLysTyrAsp           Asn
a25-a28         CCGTACGTATTATTAGGTGCTGGTCACTATAAATACGACTTTGATGGCGTAAACCGTGGT
                                                 Tyr                    Asn
a23             CCGTACGTATTATTAGGTGCTGGTCACTACAAGTATGACTTTGATGGCGTAAATCGTGGT
                                           TyrLys
a11             CCGTACGTATTATTAGGTGCTGGTCACTATAAATACGACTTTGATGGCGTAAGCCGTGGT
                                                 Tyr                    Ser
a20-a24         CCGTACGTATTATTAGGTGCTGGTCACTATAAATACGACTTTGATGGCGTAAATCGTGGT
a29, a30        CCGTACGTATTATTAGGTGCTGGTCACTATAAATACGATTTTGATGGCGTAAACCGTGGT
a31, a33        CCATACGTATTGTTAGGTGCTGGTCACTACAAGTATGACTTTGATGGCGTAAACCGTGGT
Aa involved     Pro      Leu               TyrLysTyrAsp           Asn
Type of change    t        t                 t  t  t  t             t
Syn or non-syn   Sy       Sy                Sy Sy Sy Sy            nSy
ap1             CCATACGTATTGTTAGGTGCTGGTCACTACAAGTATGACTTTGATGGCGTAAACCGTGGT
ap2             CCATACGTATTGTTAGGTGCTGGTCACTACAAGTATGACTTTGATGGCGTAAACCGTGGT
al1             CCATACGTATTGTTAGGTGCTGGTCACTACAAGTATGACTTTGATGGCGTAAACCGTGGT
ao1             CCGTATGTATTGTTAGGTGCTGGTCACTACAAATACGACTTTGATGGCGTAAACCGTGGT
                ProTyr   Leu               TyrLysTyr              Asn
Type of change    t  t     t                 t  t  t                t
Syn or non-syn   Sy Sy    Sy                Sy Sy Sy                Sy


a1-a3, a8-a19   ACACGTGGTACTTCTGAAGAAGGTACTTTAGGTAACGCTGGTGTTGGTGCTTTCTGGCGC   480
Aa involved              ThrSer                  AsnAla   Val
a5, a6, a21-23,
a25-a30         ACACGTGGTAACTCAGAAGAAGGTACTTTAGGTAACGCTGGTGTTGGTGCTTTCTGGCGC
a4, a7          ACACGTGGTAACTCAGAAGAAGGTACTTTAGGTAATGCTGGTGTTGGTGCTTTCTGGCGC
a20, a25        ACACGTGGTAATTCAGAAGAAGGTACTTTAGGTAACGCTGGTGTTGGTGCTTTCTGGCGC
a31, a32        ACACGTGGTAACTCAGAAGAAGGTACTTTAGGTAATGCGGGTGTTGGTGCTTTCTGGCGC
Aa involved              AsnSer                  AsnAla
Nt change                  Tt  T                   t  T
Syn or non-syn           nSy Sy                   Sy Sy
ap1             ACACGTGGTAACTCAGAAGAAGGTACTTTAGGTAATGCGGGTGTTGGTGCTTTCTGGCGC
ap2             ACACGTGGTAACTCAGAAGAAGGTACTTTAGGTAATGCGGGTGTTGGTGCTTTCTGGCGC
al1             ACACGTGGTAACTCAGAAGAAGGTACTTTAGGTAATGCTGGTGTTGGTGCTTTCTGGCGC
ao1             ACACGTGGTAATTCAGAAGAAGGTACTTTAGGTAACGCTGGTGTAGGTGCTTTCTGGCGC
Aa involved              AsnSer                  AsnAla   Val
Nt change                  Tt  T                   t  T     T
Syn or non-syn           nSy Sy                   Sy Sy    Sy

                                                                      EL4
a1-a15, a17-a27,
a29, a30        TTAAACGACGCTTTATCTCTTCGTACTGAAGCTCGTGCTACTTATAATGCTGATGAAGAG   540
a31             TTAAACGATGCTTTATCTCTTCGTACAGAAGCTCGTGCTACTTATAACGCTGATGAAGAG
a16, a28, a32   TTAAACGATGCTTTATCTCTTCGTACTGAAGCTCGTGCTACTTATAATGCTGATGAAGAG
Aa involved           AspAla                 Thr                  Asn
Type of change          t                      T                    t
Syn or non-syn         Sy                     Sy                   Sy
ap1             TTAAACGATGCTTTATCTCTTCGTACAGAAGCTCGTGCTACTTATAACGCTGATGAAGAG
ap2             TTAAACGATGCTTTATCTCTTCGTACAGAAGCTCGTGCTACTTATAACGCTGATGAAGAG
al1             TTAAACGATGCTTTATCTCTTCGTACAGAAGCTCGTGCTACTTATAACGCTGATGAAGAG
ao1             TTAAACGATGCATTATCTCTTCGTACAGAAGCTCGTGCTACTTATAACGCTGATGAAGAG
Aa involved           AspAla                 Thr                  Asn
Type of change          t  T                   T                    t
Syn or non-syn         Sy Sy                  Sy                   Sy


a1-a30, a32     TTCTGGAACTATACAGCTCTTGCTGGCTTAAACGTAGTTCTTGGTGGTCACTTGAAGCCT   600
a31             TTCTGGAACTATACAGCTCTTGCTGGTTTAAACGTAGTTCTTGGTGGTCACTTGAAGCCT
Aa involved                              Gly
Type of change                             t
Syn or non-syn                            Sy
ap1             TTCTGGAACTATACAGCTCTTGCTGGTTTAAACGTAGTTCTTGGTGGTCACTTGAAGCCT
ap2             TTCTGGAACTATACAGCTCTTGCTGGTTTAAACGTAGTTCTTGGTGGTCACTTGAAGCCT
al1             TTCTGGAACTATACAGCTCTTGCTGGTTTAAACGTAGTTCTTGGTGGTCACTTGAAGCCT
ao1             TTCTGGAACTATACAGCTCTTGCTGGTTTAAACGTAGTTCTTGGTGGTCACTTGAAGCCT
Aa involved                              Gly
Type of change                             t
Syn or non-syn                            Sy
```

```
a1-a18, a21-a32 GCTGCTCCTGTAGTAGAAGTTGCTCCAGTTGAACCAACTCCAGTTGCTCCACAACCACAA   660
Aa involved                                                  Ala
a19             GCTGCTCCTGTAGTAGAAGTTGCTCCAGTTGAACCAACTCCAGTTACTCCACAACCACAA
Aa involved                                                  Thr
Type of change                                               t
Syn or non-syn                                               nSy
ap1             GCTGCTCCTGTAGTAGAAGTTGCTCCAGTTGAACCAACTCCAGTTGCTCCACAACCACAA
ap2             GCTGCTCCTGTAGTAGAAGTTGCTCCAGTTGAACCAACTCCAGTTGCTCCACAACCACAA
al1             GCTGCTCCTGTAGTAGAAGTTGCTCCAGTTGAACCAACTCCAGTTGCTCCACAACCACAA
ao1             GCTGCACCTGTAGTAGAAGTTGCTCCAGTTGAACCAACTCCAGTTGCTCCACAACCACAA


a1-a3, a4-a32 GAGTTAACTGAAGACCTTAACATGGAACTTCGTGTGTTCTTTGATACTAACAAATCAAAC   720
                   Leu      Asp                                Thr
a3             GAGTTAACTGAAGACCTTAACATGGAACTTCGTGTGTTCTTTGATATTAACAAATCAAAC
Aa involved                                                  Ile
Type of change                                                t
Syn or non-syn                                               nSy
ap1             GAGTTAACTGAAGACCTTAACATGGAACTTCGTGTGTTCTTTGATACTAACAAATCAAAC
ap2             GAGTTAACTGAAGACCTTAACATGGAACTTCGTGTGTTCTTTGATACTAACAAATCAAAC
al1             GAGTTAACTGAAGATCTTAACATGGAACTTCGTGTGTTCTTTGATACTAACAAATCAAAC
ao1             GAGTTAACTGAAGACCTTAACATGGAACTTCGTGTGTTCTTTGATACTAACAAATCAAAC
                   Leu      Asp
Type of change     t          t
Syn or non-syn     Sy        Sy

a1, a3, a7, a10,
a11, a20, a22,
a23, a31, a32   ATCAAAGACCAATACAAGCCAGAAATCGCTAAAGTTGCTGAAAAATTATCTGAATACCCT   780
                            Tyr         Ile            GluLys   Ser
a2              ATCAAAGACCAATATAAGCCAGAAATCGCTAAAGTTGCTGAAAAATTATCTGAATACCCT
                            Tyr
a4-a9, a12-a19,
a21, a24-a30    ATCAAAGACCAATACAAGCCAGAAATTGCTAAAGTTGCTGAAAAATTATCTGAATACCCT
Aa involved                             Ile
Type of change                t           t
Syn or non-syn               Sy          Sy
ap1             ATCAAAGACCAATACAAGCCAGAAATCGCTAAAGTTGCTGAGAAGTTAACTGAATACCCT
ap2             ATCAAAGACCAATACAAGCCAGAAATCGCTAAAGTTGCTGAGAAGTTAACTGAATACCCT
al1             ATCAAAGACCAATACAAGCCAGAAATCGCTAAAGTTGCTGAGAAGTTAACTGAATACCCT
ao1             ATCAAAGACCAATACAAGCCAGAAATCGCTAAAGTTGCTGAGAAGTTAACTGAATACCCT
Aa involved                                            GluLys   Thr
Type of change                                           t  t   T
Syn or non-syn                                          Sy Sy   nSy


a1-12, a14-32   AACGCTACTGCACGTATCGAAGGTCACACAGATAACACTGGTCCACGTAAGTTGAACGAA   840
a13             AACGCTACTGCACGTATCGAAGGTCACACAGATAACACTGGTCCACGTAAGTTGAATGAA
                                                                   LeuAsn
Type of change                                                          t
Syn or non-syn                                                         Sy
ap1             AACGCTACTGCACGTATCGAAGGTCACACAGATAACACTGGTCCACGTAAGTTGAACGAA
ap2             AACGCTACTGCACGTATCGAAGGTCACACAGATAACACTGGTCCACGTAAGTTGAACGAA
al1             AACGCTACTGCACGTATCGAAGGTCACACAGATAACACTGGTCCACGTAAGTTGAACGAA
ao1             AACGCTACTGCACGTATCGAAGGTCACACAGATAACACTGGTCCACGTAAGTTAAACGAA
Aa involved                                                        Leu
Type of change                            t                          t
Syn or non-syn                           Sy                          Sy

a1-a32          CGTTTATCTTTAGCTCGTGCTAACTCTGTTAAATCAGCTCTTGTAAACGAATACAACGTT   900
ap1             CGTTTATCTTTAGCTCGTGCTAACTCTGTTAAATCAGCTCTTGTAAACGAATACAACGTT
ap2             CGTTTATCTTTAGCTCGTGCTAACTCTGTTAAATCAGCTCTTGTAAACGAATACAACGTT
al1             CGTTTATCTTTAGCTCGTGCTAACTCTGTTAAATCAGCTCTTGTAAACGAATACAACGTT
ao1             CGTTTATCTTTAGCTCGTGCTAACTCTGTTAAATCAGCTCTTGTAAACGAATACAACGTT
```

```
a1-6, a9-13,a16,
a20-23,a25, a31 GACGCTTCTCGTTTGTCTACTCAAGGTTTCGCTTGGGATCAACCGATTGCTGACAACAAA   960
a7,8,14,15,17-19,
24-30, a32      GATGCTTCTCGTTTGTCTACTCAAGGTTTCGCTTGGGATCAACCGATTGCTGACAACAAA
Aa change       AspAla
Type of change    t
Syn or non-syn   Sy
Aa involved     Asp
Type of change    t
Syn or non-syn   Sy
ap1             GATGCATCTCGTTTGTCTACTCAAGGTTTCGCTTGGGATCAACCGATTGCTGACAACAAA
ap2             GATGCATCTCGTTTGTCTACTCAAGGTTTCGCTTGGGATCAACCGATTGCTGACAACAAA
al1             GATGCATCTCGTTTGTCTACTCAAGGTTTCGCTTGGGATCAACCGATTGCTGACAACAAA
ao1             GATGCATCTCGTTTGTCTACTCAAGGTTTCGCTTGGGATCAACCGATTGCTGACAACAAA
Aa involved     AspAla
Type of change    t  T
Syn or non-syn   Sy Sy

a1-a32          ACTAAAGAAGGTCGTGCTATGAACCGTCGTGTATTCGCGACAATCACTGGTAGCCGTACT  1020
ap1             ACTAAAGAAGGTCGTGCTATGAACCGTCGTGTATTCGCGACAATCACTGGTAGCCGTACT
ap2             ACTAAAGAAGGTCGTGCTATGAACCGTCGTGTATTCGCGACAATCACTGGTAGCCGTACT
al1             ACTAAAGAAGGTCGTGCTATGAACCGTCGTGTATTCGCGACAATCACTGGTAGCCGTACT
ao1             ACTAAAGAAGGTCGTGCTATGAACCGTCGTGTATTCGCGACAATCACTGGTAGCCGTACT
ao2             ACTAAAGAAGGTCGTGCTATGAACCGTCGTGTATTCGCGACAATCACTGGTAGCCGTACT

a1-a23, a25-a32 GTAGTAGTTCAACCTGGTCAAGAAGCGGCAGCTCCTGCAGCAGCTCAATAA 1071
                   Val                         Gln
a24             GTAGTAGTTCAACCTGGTCAAGAAGCGGCAACTCCTGCAGCAGCTCAATAA
                                            Gln
Type of change                                t
Syn or non-syn                               Sy
ap1             GTAGTTGTTCAACCTGGTCAAGAAGCGGCAGCTCCTGCAGCAGCTCAATAA
ap2             GTAGTTGTTCAACCTGGTCAAGAAGCGGCAGCTCCTGCAGCAGCTCAATAA
al1             GTAGTTGTTCAACCTGGTCAAGAAGCGGCAGCTCCTGCAGCAGCTCAATAA
ao1             GTAGTTGTTCAACCTGGTCAAGAAGCGGCAGCTCCTGCAGCAGCTCAATAA
Aa involved        Val
Type of change       T
Syn or non-syn      Sy
```

B) Amino acid substitutions and possible effects

EL1 region

Ser52 [7 alleles including V1(lct)a1]: polar side chain, uncharged, contains hydroxyl group

Ala52 (7 alleles): hydrophobic side chain

Gly52 (18 alleles): minimal side chain (one hydrogen atom), considered polar.

In all cases the aa substitutions are described as conservative.

Between non-*baumannii* V1(lct) as compared to V1(lct)a1

Gly52 in all cases

EL2 region

Among *A. baumannii* alleles

Ala89 (30 alleles, including V1(lct)a1): hydrophobic side chain

Pro89 (2 alleles).

Intra-species amino acid change, described as conservative.

Ser90 (28 alleles, including V1(lct)a1): polar side chain, uncharged, contains hydroxyl group

Ala90 (2 alleles): hydrophobic side chain

Val90 (2 alleles): hydrophobic side chain

Intra-species amino acid changes, described as conservative.

Among non-*baumannii* alleles

Ala90 in all cases, change described as conservative.

EL3 region:
Among *A. baumannii* alleles
Asn138 (31 alleles including V1(lct)a1): polar, uncharged, amide of aspartic acid.
Ser138 (1 allele): polar, uncharged, contains hydroxyl group
Intra-species amino acid change, described as conservative.

In non-*baumannii* alleles
Asn138, no change as compared to V1(lct)a1

Among *A. baumannii* alleles
Thr144 (15 alleles, including V1(lct)a1: polar, uncharged, contains hydroxyl group
Asn144 (17 alleles): polar, uncharged
Intra-species amino acid change described as conservative.

In non-*baumannii* alleles
Asn144: polar, uncharged,
Inter-species amino acid change as compared to V1(lct)a1, described as conservative.

Non-exposed regions
At the periplasmic domain
Among *A. baumannii* alleles
Ala216 (31 alleles): hydrophobic.
Thr216 (1 allele). Polar, uncharged, contains hydroxyl group.
Change described as conservative.
In non-*baumannii* alleles
No change as compared to V1(lct)a1

Among *A. baumannii* alleles
Thr236 (31 alleles)
Ile236 (1 allele): hydrophobic, change described as conservative.

In non-*baumannii* alleles
No change as compared to V1(lct)a1

Among *A. baumannii* alleles
Ser257 (32 alleles), no change
In non-*baumannii* alleles
Thr257: polar, uncharged, contains hydroxyl group
Change described as conservative.

C) Summary of synonymous and non-synonymous substitutions at polymorphic sites detected between V1(lct) *ompA* alleles in the *Acinetobacter* population analyzed. The numbers between brackets denote the corresponding percentages among the 356 codons in each case.

Gene regions	Total changes	Synonymous changes	% syn	Non-synonymous changes	% non-syn
a) Intra-species changes (polymorphic sites found between the 32 A. baumannii V1(lct) alleles)					
All	34 (9.55)	27 (7.58)	79.4	7 (1.97)	20.6
EL regions	15 (4.21)	10 (2.81)	29.4	5 (1.40)	14.7
Non-exposed regions (total)	19 (5.34)	17 (4.76)	50.0	2 (0.56)	5.9
TM regions only	9 (2.53)	9 (2.53)	33.3	0	0
b) Inter-species changes (polymorphic sites found between A. pittii V1(lct)ap1 and A. baumannii V1(lct)a1)					
All	26 (7.30)	22 (6.18)	84.6	4 (1.12)	15.4
EL regions	10 (2.81)	7 (1.97)	26.9	3 (0.84)	11.6
Non-exposed regions	16 (4.49)	15 (4.21)	57.7	1 (0.28)	3.8
TM regions only	8 (2.25)	8 (2.25)	30.8	0	0
c) Inter-species changes (polymorphic sites found between A. lactucae V1(lct)al1 and A. baumannii V1(lct)a1)					
All	27 (7.58)	23 (6.46)	85.2	4 (1.12)	14.8
EL regions	11 (3.09)	8 (2.25)	29.6	3 (0.84)	11.1
Non-exposed regions	16 (4.49)	15 (4.21)	55.6	1 (0.28)	3.7
TM regions only	7 (1.97)	7 (1.97)	25.9	0	0
c) Inter-species changes (polymorphic sites found between A. oleivorans V1(lct)ao1 and A. baumannii V1(lct)a1)					
All	32 (8.99)	28 (7.86)	87.5	4 (1.12)	12.5
EL regions	14 (3.93)	11 (3.01)	33.4	3 (0.84)	9.4
Non-exposed regions	18 (5.01)	17 (4.78)	53.1	1 (0.28)	3.1
TM regions only	8 (2.25)	8 (2.25)	25.0	0	0

Table S3. N-terminal transit peptides and C-terminal sequences of OmpA proteins of *Acinetobacter* genus species. The *Acinetobacter* species and the accession numbers of the corresponding OmpA proteins as well as the different ecologically-differentiated clades in which the genus was recently divided (ref. 1) are indicated in the first column. In the case of ACB complex members, the corresponding OmpA variant alleles (as determined in this work) are also indicated. The accession numbers for non-*baumannii* species are indicated in Table S2. The aligned sequences at the left show the amino acid sequences of the transit peptides in each case, with the deletion/insertion of a threonine residue (Thr10) indicated by a hyphen (-) and the processing site for Signal peptidase I with a "▼" symbol (inferred using http://www.cbs.dtu.dk/services/SignalP/). The aligned C-terminal amino acid residues of the corresponding OmpAs are shown at the right.

Species and clades	Transit peptide	C-terminus
A.baylyi ADP1 CAG67610.1	MKLSRIALATMLVAAPLAAANA▼	......TVTVQPGQQAPAAQ
A.ursingii WP_004991206.1	MKLSRIALATMLVAAPFAAANA▼	......TVVVQPGQQAQ
A.soli WP_076032993.1	MKLSRIALATMLVAAPLAAANA▼	......TVTVQPGQQAQ
**Clade I (ACB complex)**		
A. baumannii		
A.baumannii V1(lct) alleles	MKLSRIALATMLVAAPLAAANA▼	......TVVVQPGQEAAAPAAAQ
A.baumannii V1(sct) alleles	MKLSRIALATMLVAAPLAAANA▼	......TVVVQPGQQAQ
A.baumannii V2(lct) alleles	MKLSRIALATMLVAAPLAAANA▼	......TVVVQPGQEAAAPAAAQ
A.baumannii V3(sct) alleles	MKLSRIALATMLVAAPLAAANA▼	......TVVVQPGQQAQ
A.baumannii V4(lct) alleles	MKLSRIALATMLVAAPLAAANA▼	......TVVVQPGQEAAAPAAAQ
A.baumannii V4(sct) alleles	MKLSRIALATMLVAAPLAAANA▼	......TVVVQPGQQAQ
A.baumannii V5(sct) alleles	MKLSRIALATMLVAAPLAAANA▼	......TVVVQPGQQAQ
**Non-*baumannii* species**		
A.pittii XJ88 V1(lct)	MKLSRIALATMLVAAPLAAANA▼	......TVVVQPGQEAAAPAAAQ
A.pittii 2012N08-034 V1(lct)	MKLSRIALATMLVAAPLAAANA▼	......TVVVQPGQEAAAPAAAQ
A.lactucae CI78 V1(lct)	MKLSRIALATMLVAAPLAAANA▼	......TVVVQPGQEAAAPAAAQ
A.oleivorans DR1_DE0008 V1(lct)	MKLSRIALATMLVAAPLAAANA▼	......TVVVQPGQEAAAPAAAQ
A.pittii AB17H194 V1(sct)	MKLSRIALATMLVAAPLAAANA▼	......TVVVQPGQQAQ
A.pittii AP007 V1(sct)	MKLSRIALATMLVAAPLAAANA▼	......TVVVQPGQQAQ
A.nosocomialis 6411 V1(sct)	MKLSRIALATMLVAAPLAAANA▼	......TVVVQPGQQAQ
A.seifertii MI30-324 V1(sct)	MKLSRIALATMLVAAPLAAANA▼	......TVVVQPGQQAQ
A.seifertii TUM15279 V1(sct)	MKLSRIALATMLVAAPLAAANA▼	......TVVVQPGQQAQ
A.seifertii SAb133 V1(sct)	MKLSRIALATMLVAAPLAAANA▼	......TVVVQPGQQAQ
A.calcoaceticus ANC3811 V2(lct)	MKLSRIALA-MLVAAPLAAANA▼	......TVVVQPGQEAAAPAAAQ
A.oleivorans KCJK7897 V2(lct)	MKLSRIALA-MLVAAPLAAANA▼	......TVVVQPGQEAAAPAAAQ
A.nosocomialis WC-487 V3(sct)	MKLSRIALATMLVAAPLAAANA▼	......TVVVQPGQQAQ
A.calcoaceticus RUH2202 V3(sct)	MKLSRIALA-MLVAAPLAAANA▼	......TVVVQPGQQAQ
A.pittii AP_882 V3(sct)	MKLSRIALA-MLVAAPLAAANA▼	......TVVVQPGQQAQ
A.oleivorans DR1/DR1 V3(lct)	MKLSRIALA-MLVAAPLAAANA▼	......TVVVQPGQEAAAPAAAQ
A.pittii PHEA-2 V3(lct)	MKLSRIALA-MLVAAPLAAANA▼	......TVVVQPGQEAAAPAAAQ
A.lactucae ANC4052 V3(lct)	MKLSRIALA-MLVAAPLAAANA▼	......TVVVQPGQEAAAPAAAQ
A.calcoaceticus ANC3680 V3(lct)	MKLSRIALA-MLVAAPLAAANA▼	......TVVVQPGQEAAAPAAAQ
A.pittii ATCC 19004 V5(sct)	MKLSRIALA-MLVAAPLAAANA▼	......TVVVQPGQQAQ
A.pittii ANC3678 V5(sct)	MKLSRIALA-MLVAAPLAAANA▼	......TVVVQPGQQAQ
A.pittii WCHAP005046 V5(lct)	MKLSRIALA-MLVAAPLAAANA▼	......TVVVQPGQEAAAPAAAQ
**Clade II**		
A.junii WP_039047009.1	MKLSRIALA-MLVAAPLAAANA▼	......TVLVQPGQQAQ
A.beijerinckii WP_005061574.1	MKLSRIALA-MLVAAPLAAANA▼	......TVLVQPGQQAQ
A.parvus WP_050041456.1	MKLSRIALA-MLVAAPLAAANA▼	......TVLVQPGQQAQ
A.proteolyticus WP_101235588.1	MKLSRIALA-MLVAAPLAAANA▼	......TVLVQPGQQAQ
A.haemolyticus SPT46410.1	MKLSRIALA-MLVAAPFAAANA▼	......TVLVQPDQQAQ

```
A.venetianus WP_019383768.1      MKLSRIALA-MLVAAPLAAANA▼......TVLVQPGQ
A.tjernbergiae WP_018677830.1    MKLSRIALA-MLVAAPLAAANA▼......TVLVQPGQ

Clade III
A.gerneri WP_004854794.1         MKLSRIALA-MLVAAPLAAANA▼......TVVQDAQ
A.bereziniae WP_004829936.1      MKLSRIALA-MLVAAPLAAANA▼......TVTKTVTK
A.rudis EPF79807.1               MKLSRIALA-MLVAAPLAAANA▼......TVLAQPRAQPR
A.indicus AQU14364.1             MKMSRIALA-MLVAAPLAAANA▼......TVTVQPEAAAQ
A.schindleri WP_076754170.1      MKMSRIALA-MLVAAPLAAANA▼......TVLAEQPAAQ
A.lwoffii AUC08284.1             MKMSRIALA-MLVAAPFAAANA▼......TVLAEQPAQ
A.bouvetii WP_005007826.1        MKMSRIALA-MLVAAPLAAANA▼......TVVVEGQQAQ
A.johnsonii WP_058870218.1       MKMSRIALA-MLVAAPLAAANA▼......TVQAQ
A.nectaris WP_023273734.1        MKLSRIALATVLAASPFVVANA▼......TVLAQPKAQ
A.brisouii WP_045794742.1        MKLSRIAVATLLAASPLVAANA▼......TVIAQPTAPAAQ
```

Table S4: Variation in substitution patterns between extracellular loop regions and non-exposed regions of *ompA* for total alignments, and alignments where regions identified as recombinant using RDP have been removed (Recombination removed – only detected in V2 alleles). [a]Values for non-exposed protein domains ($\kappa1$ and $\omega1$) and exposed extracellular loops ($\kappa2$ and $\omega2$) are shown. κ, transition/transversion rate ratio; ω, d_N/d_S ratio; LRT, likelihood ratio test. [b]Significance assessed using χ^2 tests, with *p* value corrected for multiple testing using a Bonferroni correction.

***ompA* clades**	**Fixed parameter model**		**Variable parameter model[a]**		**LRT statistic**	***p* value[b]**
	κ	ω	κ	ω		
V1	3.78688	0.06912	$\kappa1$: 2.88613	$\omega1$: 0.12152	29.41	<0.00001
			$\kappa2$: 6.34718	$\omega2$: 0.01875		
V2	5.77821	0.02889	$\kappa1$: 5.67814	$\omega1$: 0.0349	13.75	0.00104
			$\kappa2$: 999	$\omega2$: 0.0001		
V4	3.26652	0.04342	$\kappa1$: 3.64483	$\omega1$: 0.04378	7.84	0.01984
			$\kappa2$: 2.57935	$\omega2$: 0.04206		
Recombination removed						
V2	4.15706	0.10886	$\kappa1$: 1.808	$\omega1$: 0.28346	18.69	0.00009
			$\kappa2$: 999	$\omega2$: 0.0001		

Table S5: Bacteria with close matches to the 18-nucleotide high-GC sequence coding for an alanine-rich tract located at the c-terminal end of some OmpA variants.

Phyla	Species	GenBank Accession number
Actinobacteria	*Streptomyces* sp. HNM0039	CP029188
	Propionibacterium australiense	LR134442
	Gordonia spp.	CP022580, CP002907
	Pseudonocardia spp.	CP010989, CP012181, CP012184
	Stackebrandtia nassauensis	CP001778
Bacteroidetes	*Hymenobacter* APR13	CP006587
Proteobacteria	*Pseudomonas stutzeri*	CP007441
	Serratia ficaria	LT906479
	Comamonas spp.	LN879547, CP001220
	Bradyrhizobium canariense	LT629750
	Martelella endophytica	CP010803
	Novosphingobium THN1	CP028347
